# Microarray and comparative genomics-based identification of genes and gene regulatory regions of the mouse immune system

**DOI:** 10.1186/1471-2164-5-82

**Published:** 2004-10-25

**Authors:** John J Hutton, Anil G Jegga, Sue Kong, Ashima Gupta, Catherine Ebert, Sarah Williams, Jonathan D Katz, Bruce J Aronow

**Affiliations:** 1 Department of Pediatrics and Biomedical Informatics, University of Cincinnati and Cincinnati Children's Hospital Research Foundation, Cincinnati, Ohio 45229, USA

## Abstract

**Background:**

In this study we have built and mined a gene expression database composed of 65 diverse mouse tissues for genes preferentially expressed in immune tissues and cell types. Using expression pattern criteria, we identified 360 genes with preferential expression in thymus, spleen, peripheral blood mononuclear cells, lymph nodes (unstimulated or stimulated), or *in vitro *activated T-cells.

**Results:**

Gene clusters, formed based on similarity of expression-pattern across either all tissues or the immune tissues only, had highly significant associations both with immunological processes such as chemokine-mediated response, antigen processing, receptor-related signal transduction, and transcriptional regulation, and also with more general processes such as replication and cell cycle control. Within-cluster gene correlations implicated known associations of known genes, as well as immune process-related roles for poorly described genes. To characterize regulatory mechanisms and cis-elements of genes with similar patterns of expression, we used a new version of a comparative genomics-based *cis*-element analysis tool to identify clusters of *cis*-elements with compositional similarity among multiple genes. Several clusters contained genes that shared 5–6 *cis*-elements that included ETS and zinc-finger binding sites. *cis*-Elements AP2 EGRF ETSF MAZF SP1F ZF5F and AREB ETSF MZF1 PAX5 STAT were shared in a thymus-expressed set; AP4R E2FF EBOX ETSF MAZF SP1F ZF5F and CREB E2FF MAZF PCAT SP1F STAT cis-clusters occurred in activated T-cells; CEBP CREB NFKB SORY and GATA NKXH OCT1 RBIT occurred in stimulated lymph nodes.

**Conclusion:**

This study demonstrates a series of analytic approaches that have allowed  the implication of genes and regulatory elements that participate in the  differentiation, maintenance, and function of the immune system.  Polymorphism or mutation of these could adversely impact immune system  functions.

## Background

The immune system is composed of a multiplicity of individual cell types that derive from a relatively small number of immuno-hematopoietic progenitors that undergo complex developmental and exposure-driven differentiation and activation. Cell-type specific gene expression is driven to a large measure by complex transcriptional regulation that orchestrates differential expression of a wide variety of genes necessary to accomplish immune effector functions. A number of specific transcription factors (TFs) which regulate gene expression in immune system cell types have been identified, largely through gene knockout experiments and isolation of protein complexes that bind to regulatory regions of target genes. Examples include PU.1/Ets, Ikaros, E2A, EBF, PAX5, GATA3, NFAT, cMYB, and OCT-2 [1-4]. These proteins bind to clusters of *cis*-regulatory elements in multiple diverse combinations to give rise to specific patterns of gene expression [5]. However, the layout of regulatory and coding regions is not known for most genes that are preferentially expressed in lymphocytes and immune tissues (see, for examples, [6-11]). Based on the nearly completed nucleotide sequences of the mouse and human genomes ([12]; [13]), we have sought to expand our knowledge of the structure and function of compartment-specific genes, and in particular, to find clusters of *cis*-elements that bind TFs and regulate gene expression during biological processes. DNA sequences of both coding regions and non-coding regions which harbor *cis*-elements that govern expression, are phylogenetically conserved [14-16]. This conservation of functionally important regions of DNA underpins current methods of identifying putative regulatory regions by comparative sequence analysis. In practice, finding relevant clusters of *cis*-elements is difficult and computationally intensive.

High-throughput gene expression profiling provides a powerful approach to the investigation of relative transcriptional activity as a function of biological differentiation across a variety of cells and tissues. Published examples that probe a wide variety of distinct, differentiated materials include the Human Gene Expression (HuGE) Index database [17] and the GNF (Genomics Institute of the Novartis Research Foundation; ) database of human and mouse gene expression [18]. These resources provide access to patterns of expression of a significant fraction (15–25%) of all mouse and human genes in several dozen tissues and cell types. We have created a large database locally, which has permit investigators from our campus to profile gene expression in mouse tissues and cell types specific to their interests [19]. To do this, we used the Incyte Mouse GEM1 microarray, an 8638 element spotted cDNA gene expression platform and a universal reference design that employed poly A+ mRNA was prepared from whole day-1 postnatal mouse. Two channel Cy3-Cy5 microarray hybridization technology was used to identify relative strength of signals from each element of the array for a specific tissue. From this database, we identified 360 cDNAs on the microarray that exhibit preferential expression in immune tissues such as lymph nodes, thymus, and activated T-cells relative to most other types of tissues. We identified 333 genes that encode these sequences and have grouped them by biological functions and by patterns of expression.

*Cis*-element clusters that are conserved in pairs of orthologs are strong predictors of regulatory regions within mammalian genes [15,20,21]. We have used this method to identify putative regulatory modules, which are clusters of conserved *cis*-regulatory elements that occur in coordinately regulated genes of the immune system and may play a role in controlling their expression during development or mature cell function. Several of the modules identified through this approach contain *cis*-elements whose biological relevance has been experimentally validated in previous studies. Other computationally identified modules from this immunomic database have not been studied in detail, but the results and a tool to analyze them further, are provided at the website [22]. Taken together these data provide valuable guidance to the design of experiments that seek to identify regulatory modules in genes with specific patterns of expression.

## Results

### Selection of set of immune genes

Our goal is to identify genes, which are essential for the differentiation, maintenance, and function of the immune system, and their associated regulatory elements. Polymorphisms or mutation in these might underlie well-known variation among individuals in effectiveness of their immune response. Mouse immune genes were identified from our gene expression database constructed using the 8638 element microarray and probed with mRNA prepared from 65 normal adult and fetal tissues. We chose to select relevant genes by collecting those expressed above a threshold value rather than by statistical analysis of variance. Given the small number of replicates and the large number of comparisons being made, we would not have enough statistical power to detect differentially expressed genes by using traditional statistical tests with appropriate specificity. In addition, with the reduced specificity of statistical tests, the biologically non-significant, but somewhat reproducible differences in gene expression will obscure changes that are of biologically significant magnitude, but vary from replicate to replicate. Expression of genes is not discontinuous from tissue to tissue, but varies quantitatively over a wide range. The threshold to distinguish expressed from non-expressed genes was set to identify the hundred or so most highly expressed gene in each relevant tissue.

**Figure 1:**
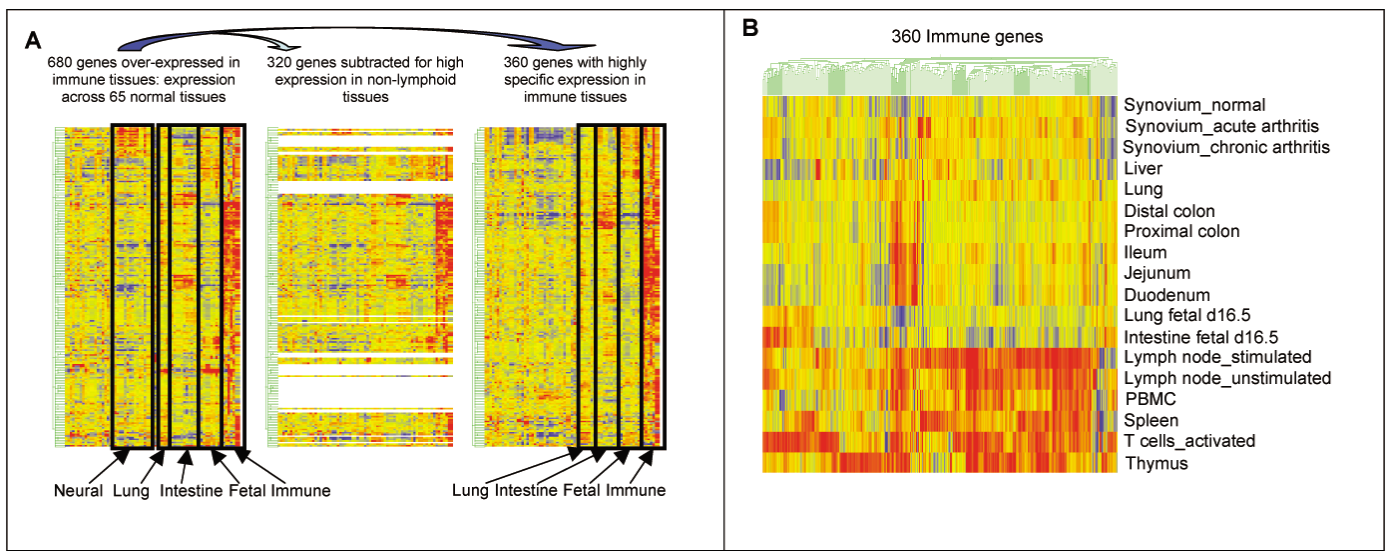
**Expression profiles of sequences across tissues: **Hierarchical tree clustering of genes and tissues was carried out using Pearson correlation and the log of the average of the relative expression ratio for each gene, as measured in replicate arrays. Sequences with similar expression patterns across all tissues are clustered together in the resulting trees, the closeness of the sequences in sub trees is a measure of how closely correlated their expression is. (A) Hierarchical tree clustering of genes across 65 normal adult and fetal tissues. 680 sequences were identified that were highly expressed in thymus, unstimulated and stimulated lymph nodes, spleen, peripheral blood mononuclear cells, and *in vitro *activated T-cells. To increase specificity, 320 sequences were removed because they were also highly expressed in one or more non-lymphoid tissues, as described in the text. The pattern of expression of the remaining 360 "immune genes" across tissues is shown. (B) Hierarchical tree clustering of 360 immune genes across 18 normal adult and fetal tissues. There are 3 major groups of tissues that show clusters of highly expressed "immune genes" These include the 6 immune tissues, various segments of adult intestine, and fetal day 16.5 lung and intestine. Less prominent clusters are seen in adult lung and liver. Genes in these clusters are described in the text. While the band of high expression extends across all genes for the 6 immune tissues, relative expression of each gene within the immune tissues shows distinct patterning.

Genes were considered to be "immune genes" if they were more highly expressed in one or more of 6 immune tissues (lymph nodes from normal and antigen stimulated mice, thymus, activated T-cells, spleen, peripheral blood mononuclear cells) than in most other normal adult and fetal mouse tissues. 680 genes were identified where the amount of cDNA hybridized from one or more immune tissues was 3 or more times greater than hybridization of cDNAs from the reference whole mouse (Figure 1A). To increase specificity, the 680 genes were then filtered to remove those with 2-fold or greater expression in normal brain, spinal cord, heart, kidney, pancreas or stomach. These tissues were chosen because they do not play a role in the immune response, contain very few cells of the immune system, and should not express immune-specific genes. By contrast, no effort was made to remove genes expressed in the intestinal tract, lung, or fetus where cells of the immune system might be expected. The resulting set of 483 genes was examined by hierarchical cluster analysis. Spleen and peripheral blood mononuclear cells were noted to express genes encoding proteins of immature erythroid cells and polymorphonuclear leukocytes. To remove these, the set was restricted to genes that were expressed 2 fold or greater in at least one of stimulated and unstimulated lymph nodes, activated T cells, or thymus. The end result is a set of 360 expressed sequences, which we call "immune" genes (Figure 1A and 1B). 265 of the expressed sequences were linked to specific genes and gene symbols, using the Mouse Genome Database (MGD) [23] and NCBI-LocusLink [24]. The remainders were analyzed using MouseBLAST and BLAT [12] to find sequence homologies with known genes. An additional 78 sequences could be linked to specific genes, 9 (seven occurring twice and one occurring thrice) of these were redundant, so that a total of 333 previously known unique genes were represented by the 360 expressed sequences. 292 of these genes were assigned a probable function, using criteria described in Methods. 5 sequences were repetitive elements and 12 sequences could not be linked to a known gene or function. Gene symbols, names, functions, and extensive additional annotations are provided in the supplementary materials (Additional file 1). Human orthologs of these mouse immune genes were sought by sequence homology. Where found, pairs of mouse-human orthologs were annotated with regard to function and were analyzed for phylogenetically conserved regulatory regions.

### Hierarchical clustering of genes and tissues

Hierarchical tree clustering of the 360 sequences and 65 normal adult and fetal tissues was carried out by Pearson correlation using the log of the average of the relative expression ratio for each gene as measured in replicate arrays (Figure 1A). While the band of high expression extends across the 6 immune tissues, relative expression of each gene within the immune tissues shows distinct patterning (Figure 1B). For intestinal and fetal tissues, the areas of high expression are localized and do not include the majority of the immune genes. Function of the genes expressed in these tissues will be described.

### Function of the immune genes

A putative function could be assigned to 298 expressed sequences (Additional file 1) based on one or more known functional annotation or sequence analysis-based structural classifiers. This annotation is independent of pattern of expression and gives an overview of the types of functions carried out by immune genes. Six functional groups derived from these annotations are shown in Table 1. The HGNC [25] and MGI [23] approved gene symbols are used in the table, although many of these genes are better known by their aliases as provided in supplementary materials. Table 1 shows 59 genes that have functions associated with defense-immune or defense response (immune is a subcategory of defense in GO annotations). Defense-immune genes were more directly related to antigen recognition and receptor signaling of T- and B-lymphocytes than defense genes, although the separation of defense and immune is somewhat arbitrary. 47 genes in Table 1 are involved in cell signaling, 14 in apoptosis, 8 in chemotaxis, and 6 in lysosomes. Additional lists of genes grouped by function and shown in the supplementary materials include 39 in transcription, 23 in DNA replication/cell cycle control, 20 in protein synthesis, 13 in transport, and 10 in adhesion. Smaller groups of genes that are important in function of the immune system include protein trafficking and degradation, and maintenance of the cytoskeleton. Functions carried out by some of the genes that are highly expressed in immune tissues are common to cells and tissues that are actively proliferating and synthesizing proteins. These include, for example, genes involved in DNA synthesis and the cell cycle such as the minichromosome maintenance proteins, *Mcm2 *through *Mcm7*; the DNA polymerases and primase, *Pola2*, *Cdc6*, *Prim1*; the processivity factor *Pcna*, and cyclin E1, *Ccne1*. They play a role in regulation of chromosomal replication in many types of cells [26]. In the immune tissues, high expression of these genes is characteristic of activated T-cells, which are proliferating. Similarly, other immune genes are involved in protein synthesis and are not specific to the immune system. Twelve immune genes encode ribosomal proteins.

**Table 1:** Six sets of genes that are highly expressed in immune tissues, grouped by function. Gene symbol and GenBank accession number identify genes

**Defense – Immune – 38 Genes**		**Defense – 21 Genes**		**Signal – 47 Genes**	
*1190001G19Rik*	NM_026875	*5830443L24Rik*	BC031475	*1200013B08Rik*	NM_028773
*A630096C01Rik*	BB629669	*Arl6ip2*	BC006934	*2410118I19Rik*	AK004869
*AI789751*	AI789751	*Bst1*	NM_009763	*2610207I05Rik*	AK011909
*B2m*	NM_009735	*C1qg*	NM_007574	*Adcy7*	NM_007406
*BB219290*	NM_145141	*C1s*	NM_144938	*AI325941*	BF181435
*Btla-interim*	BM240873	*Camp*	NM_009921	*Arhh*	AK017885
*Cd14*	NM_009841	*Daf1*	NM_010016	*Cd37*	NM_007645
*Cd79b*	NM_008339	*Gbp2*	NM_010260	*Cd53*	NM_007651
*Cd86*	BC013807	*Gzmb*	NM_013542	*Cd97*	NM_011925
*Cxcl9*	NM_008599	*Klra24-pending*	AA288274	*Clecsf12*	NM_020008
*Fcgr3*	NM_010188	*Klrd1*	NM_010654	*Clecsf5*	NM_021364
*Gp49a*	NM_008147	*Ncf2*	NM_010877	*Clk3*	AF033565
*H2-Aa*	NM_023145	*Ncf4*	NM_008677	*Coro1a*	NM_009898
*H2-Ab1*	NM_010379	*Oas2*	NM_145227	*D530020C15Rik*	BC027196
*H2-DMa*	NM_010386	*Oasl2*	NM_011854	*Dgkz*	BC014860
*H2-Eb1*	NM_010382	*Ocil-pending*	NM_053109	*Dok2*	NM_010071
*H2-K*	U47328	*Prg*	NM_011157	*E430019B13Rik*	AA881918
*H2-L*	M34961	*Tnfrsf13b*	AK004668	*G431001E03Rik*	AA387272
*H2-Oa*	NM_008206	*Tnfrsf4*	NM_011659	*Gnb2-rs1*	NM_008143
*H2-Ob*	NM_010389	*Tnfrsf9*	NM_011612	*Gpcr25*	NM_008152
*H2-Q7*	NM_010394	*Zbp1*	AA175243	*Gprk6*	NM_011938
*Igh-4*	L36938			*Hck*	NM_010407
*Igj*	BC006026	**Apoptosis – 14 Genes**		*Iigp-pending*	NM_021792
*Igl*	AK008551			*Il2rg*	NM_013563
*Igsf7*	AF251705	*5630400E15Rik*	AK017464	*Il4ra*	NM_010557
*Lst1*	AF000427	*AI447904*	BF179348	*Jak1*	BC031297
*Ly86*	NM_010745	*Axud1*	BC029720	*Lck*	BC011474
*Mpa2*	NM_008620	*Biklk*	BC010510	*Lcp2*	BC006948
*Mpeg1*	L20315	*Birc2*	NM_007464	*Lyn*	BC031547
*Ms4a1*	NM_007641	*Casp4*	NM_007609	*Lypla1*	BF160555
*Ms4a4b*	NM_021718	*Dnase1l3*	NM_007870	*Map3k1*	AF117340
*Ms4a6c*	NM_028595	*Ian4*	NM_031247	*Map4k1*	BC005433
*Sema4d*	NM_013660	*Ifi203*	AA174447	*Mbc2*	BC011482
*Tactile-pending*	NM_032465	*Ripk3*	NM_019955	*P2y5*	AK011967
*Tcrd*	AI530748	*Scotin-pending*	NM_025858	*Pilra*	AJ400844
*Tcrg*	NM_011558	*Stk17b*	NM_133810	*Pip5k2a*	AK012196
*Tlr1*	NM_030682	*Stk4*	W77521	*Ptpn2*	NM_008977
*Trygn16*	M97158	*Trp53inp1*	NM_021897	*Ptpn8*	NM_008979
				*Ptprc*	NM_011210
**Lysosomes – 6 Genes**		**Chemotaxis – 8 Genes**		*Ptprcap*	NM_016933
				*Rac2*	NM_009008
*Acp5*	AA002801	*Ccl19*	NM_011888	*Stat1*	NM_009283
*Ctsl*	NM_009984	*Ccl22*	NM_009137	*Stat3*	BC003806
*Ctss*	NM_021281	*Ccl4*	NM_013652	*Stat4*	NM_011487
*Ctsz*	NM_022325	*Ccl6*	NM_009139	*Stk10*	NM_009288
*Man1a*	NM_008548	*Ccr2*	NM_009915	*Syk*	NM_011518
*Man2b1*	NM_010764	*Cxcl13*	NM_018866	*Tln*	NM_011602
		*Cxcr4*	NM_009911		
		*S100a8*	NM_013650		

There are sets of genes that work together to produce the cellular and humoral immune responses. For example, molecules of the major histocompatibility complex present foreign peptides to T cells. They are encoded by genes such *H2-Aa*, *H2-Ab1*, *H2-DMa*, *H2-Eb1*, *H2-K*, *H2-L*, *H2-Oa*, *H2-Ob*, *H2-Q7*, *and B2m *(Table 1, Defense – Immune). Signal transduction pathways are abundant and play critical roles in the function of lymphocytes. They link the recognition of antigens or chemokines by receptors on the cell surface to the transcription of genes required for cell division and new protein synthesis. This process of lymphocyte activation requires an intracellular signaling cascade with participation of protein kinases, G-proteins, and products of cleavage of membrane phospholipids [27-29] (Table 1, Signal). Janus kinases, encoded by genes such as *Jak1*, phosphorylate both signal transducers and activators of transcription (*Stat1*, *Stat3*, and *Stat4*) as part of the lymphocytes' response to cytokines. The product of *Rac2 *is a G protein that participates in the cascade of kinases leading to activation of TFs. Chemokines are a family of small proteins that activate cells such as lymphocytes as part of the host response to infection. Genes that encode the chemokines (*Ccl4*, *Ccl6*, *Ccl19*, *Ccl22*, *Cxcl13*) and chemokine receptors (*Cxcr4 *and *Ccr2*) (Table 1, Chemotaxis) are highly expressed in immune tissues.

Twenty-one sequences representing 19 known genes were highly expressed in gastrointestinal tissue (Figure 1B). Of these, 5 were classified as "Defense – Immune", including *B2m*, *H2-Q7*, *Tcrg*, *Tlr1*, and *H2-K*. Of the 51 genes expressed in fetal tissues (Figure 1B), 46 are annotated. Sixteen genes functioned in protein synthesis and 13 in cell cycle/DNA synthesis. No "Defense" or "Defense-Immune" genes were highly expressed in fetal tissues. Genes expressed in fetal tissues reflect active growth and proliferation of cells. In immune tissues, these same genes are particularly well expressed in activated T-cells and thymus, where cell proliferation is occurring.

### Cis-regulatory elements of MHC class I genes

**Figure 2:**
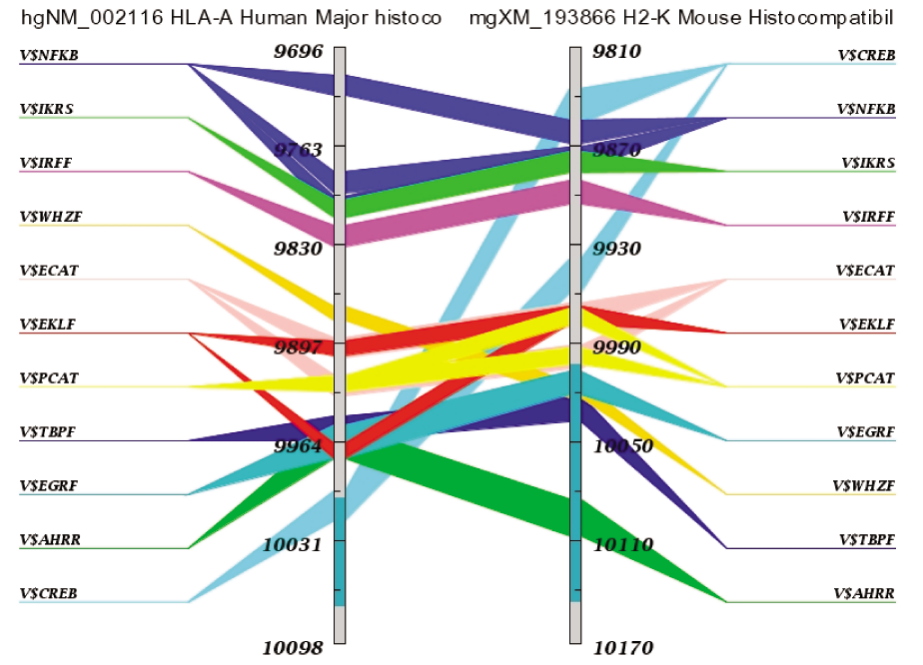
**Computationally predicted clusters of *cis*-elements in the promoter region of mouse *H2-K *and its human ortholog *HLA-A***: The ATG of human *HLA-A *is at position 10,001 while that of mouse *H2-K *is at 10,463. Thus, the region represented relative to ATG is -305 to +97 (human) and -653 to -293 (mouse). Additionally, these regions correspond to chr 6: 30,015,866–30,016,268 (+) of the Human Genome July 2003 Assembly and chr17: 33,638,839–33,639,199 (-) of the Mouse October 2003 Assembly . Families of transcription factor binding sites and the relative positions of the sites in the nucleotide sequences of the two genes are represented as different colored bars stretching across the ortholog gene pair.

Regulatory modules predicted by comparative analyses of DNA sequences must be validated by genomic footprinting and other biochemical techniques, which prove that the predicted *TF *binding sites are biologically relevant. Because extensive data are available, we compared the structure of the promoter elements of the *H2-K *and *HLA-A *genes (MHC class I) as predicted by computational and biochemical studies. Experimentally identified, conserved, regulatory elements within the promoter of MHC class I genes include: an enhancer A element (two NFKB sites), an interferon-stimulated response element (ISRE), site α (cAMP-response element), enhancer B (inverted CCAAT), CCAAT, and TATA elements [30]. Computationally predicted arrangements of conserved *cis*-elements in the promoters of *H2-K *and its human ortholog, *HLA-A*, are shown in Figure 2. FASTA sequences and corresponding coordinates of the regions used in the analysis are given in Additional file 10. The predicted arrangements are in close agreement with results of genomic footprinting, electrophoretic mobility shift assays, and other techniques. For *HLA-A*, computationally identified binding sites previously found by biochemical analyses include: IRFF, CREB, ECAT, PCAT, TBPF and two NFKB. The enhancer-A element of the MHC class I promoter encompasses two NFKB binding sites and plays an important role in the constitutive and cytokine-induced expression of MHC class I genes. Our IRFF site is the reported ISRE and can bind interferon regulatory factor 1 to activate MHC class I transcription. Site α of the MHC Class I promoter corresponds to our CREB binding site and plays an important role in regulation of expression of Class I genes. Our PCAT and ECAT sites include sequences consistent with the CCAAT site and our TBPF is a TATA binding site, as reported in the MHC class I promoter immediately upstream of the transcription start site [30]. Computational analysis identifies additional potential binding sites that have not yet been tested for biological relevance. These include families IKRS, WHZF, EKLF, EGRF, and AHRR. Several of these may play a specific role in the immune system. For instance, the IKRS family of sites bind Ikaros zinc finger transcription factors, which are regulators of lymphocyte differentiation; the WHZ family of TFs includes members that are critical to the proper expression of genes during development of the thymus [31]; the EGR family of zinc finger transcription factors is induced as a consequence of activation of the mitogen-activated protein kinase (MAPK) signaling pathway during positive selection in the thymus [32]; and AhR is known to effect immunosuppression by inducing bone marrow stromal cells to deliver a death signal to lymphocytes [33]. We conclude that computational analyses both identify previously reported TF binding sites and predict phylogenetically conserved sites that should be examined for biological relevance in future biochemical studies.

### Cis-Regulatory elements in genes grouped by patterns of expression

**Figure 3:**
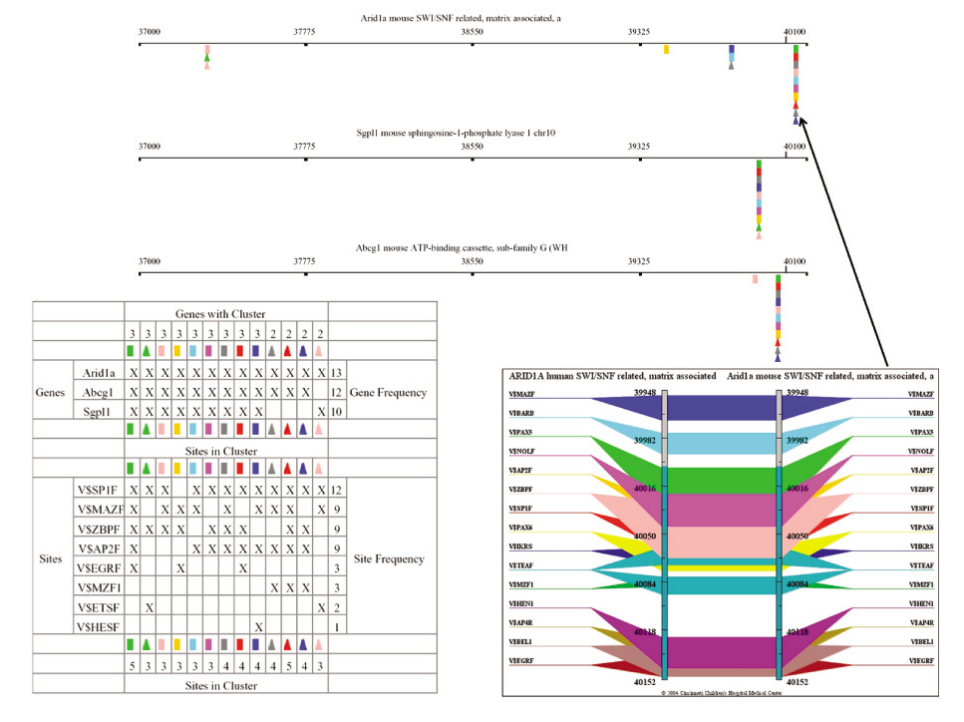
**Example of a CisMols display of location and composition of clusters of *cis*-elements that are putative regulatory modules. **The genes are those with high expression in thymus. The algorithm used by TraFac and CisMols to display regulatory modules uses a moving 200 bp window to scan regions of DNA for specific sequences characteristic of TF binding sites (*cis*-elements). Clusters of these *cis*-elements are not generally distributed evenly across a segment of DNA, but are highly localized to specific segments which are likely to play a role in regulation of gene expression. Because the scanning window is limited to 200 bp and the scan changes the frame of sequences within the window, a regulatory module that contains multiple *cis*-elements may not be displayed as one list of multiple elements, but rather as a list of several modules of different composition and arrangement within one small segment of DNA. Each colored cube indicates a cluster of 3 or more *cis*-elements with at least one "lymphoid element". The region searched is upstream 3 kb and downstream 100 bp of transcription start site (as defined by the respective mRNAs from NCBI's RefSeq database). The legend in the lower left half indicates the composition of each of the modules and the genes that share them. In the lower right hand panel is the Trafac image of one of the *cis*-element dense region with multiple shared modules of *Arid1a *gene.

Locally developed programs, TraFaC and CisMols, were used to identify and display putative regulatory modules in genes grouped by patterns of expression. The algorithms use a moving 200 bp window to scan regions of DNA for specific sequences characteristic of TF binding sites (Figure 3).

Cluster analysis of genome wide expression data from microarrays permits the grouping together of genes with similar patterns of expression across cells, tissues or experimental conditions. Clustering of genes by patterns of expression was first applied on a large scale to yeast [34], where control of important variables like genotype, phase of cell cycle, and growth conditions permits precise identification of coordinately regulated genes. Clustering has also been used to catalog mammalian genes that are differentially expressed in normal and malignant immune cells [35,36]. While yeast genes with similar patterns of expression have been found to share regulatory elements [37], identification of such elements in clustered genes of mammals is complex and not very successful [38,39]. Conservation of functionally important regions of DNA underpins current methods of identifying putative regulatory regions by computational analysis of nucleotide sequences [14-16]. Using K-means clustering in GeneSpring (Version 4.2.1), 160 genes, which had been annotated using SOURCE [40] early in our studies, were divided into distinct sets based on similarity of expression patterns across 15 tissues. Tissues were given equal weight, the number of clusters was set at 20, and similarity was measured by standard correlation. For technical reasons, GeneSpring did not assign 4 genes to clusters. The cluster sets are shown in Additional file 2.

#### K-cluster set 15

**Figure 4:**
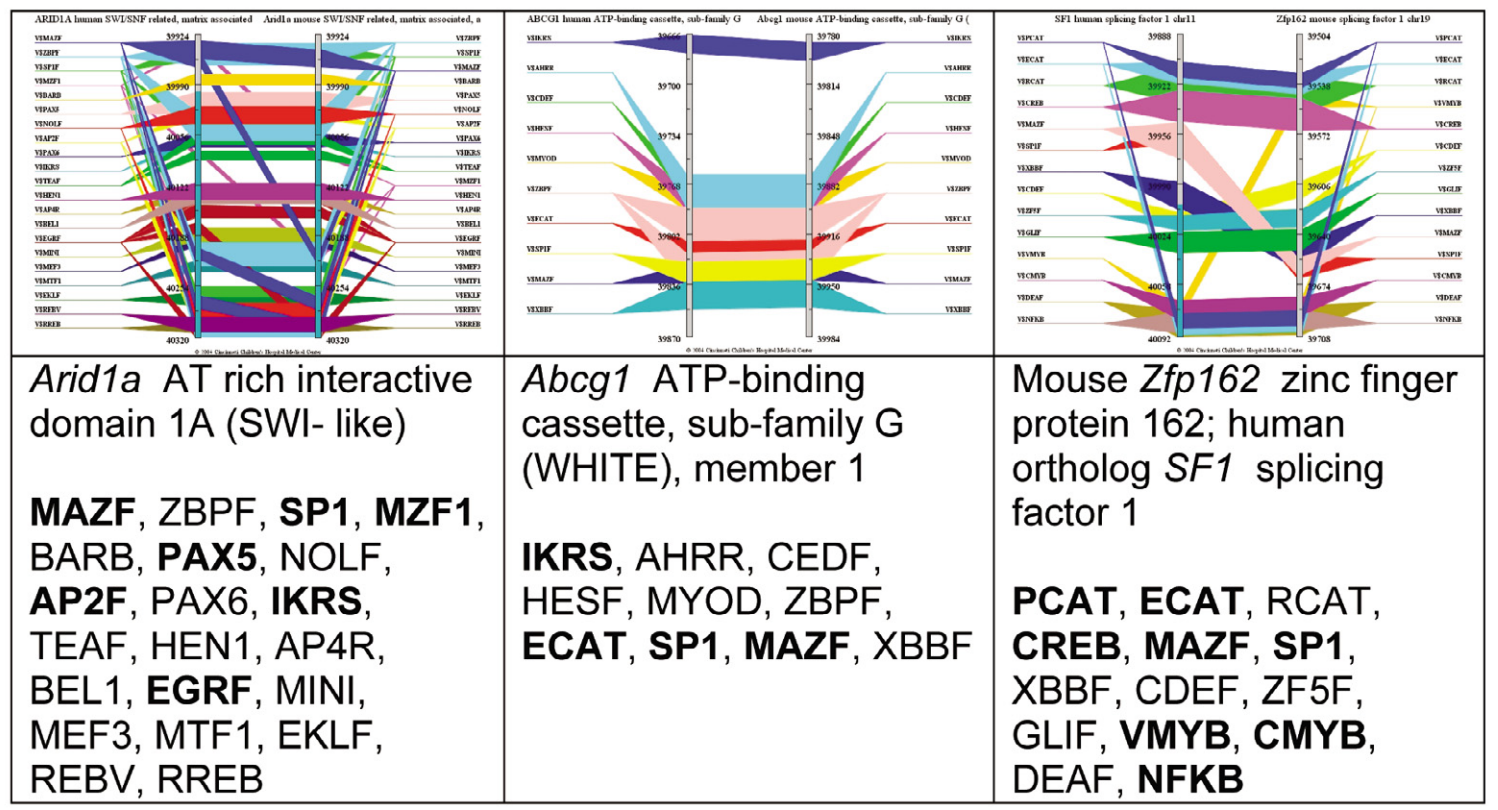
**Clusters of TF binding sites immediately upstream of the transcription start site in 3 genes of cluster set 15 (co-expressed genes with high expression in thymus)**: The upper panels compare the location of TF binding sites surrounding and upstream regions of transcription start site (based on the corresponding mRNA annotations from NCBI's RefSeq database) of human and mouse *Arid1a*, *Abcg1 *and *Zfp162 *genes in GenomeTrafac database . The bottom panels list the gene descriptions and each of the binding sites in their order of occurrence from distal (top) to proximal (bottom) to exon 1 of the human gene, which is on the left within each panel. Binding sites in bold are known "lymphoid elements". The first nucleotide of exon 1 is at bp 40,001. Each of the colored bars represents a class of TF binding sites and connects homologous binding sites in genes of the two species. The orthologous genes may differ in the number and location of specific TF's binding sites. The corresponding coordinates of the regions on human (NCBI Build 35, May 2004) genome assembly are: chr1: 26,706,590–26,706,986 (+), chr21: 42,512,113 42,512,317 (+) and chr11: 64,302,720–64,302,924 (-) for human *ARID1A*, *ABCG1 *and *SF1 *respectively. Coordinates in the mouse genome assemblies (Build 33 Mouse Assembly, May 2004) are: chr4: 132,206,952–132,207,348 (-), chr19: 6,151,958–6,152,162 (+) and chr17: 29,663,342–29,663,546 (+) for *Arid1a*, *Abcg1 *and *Zfp162 *respectively.

K-cluster set 15 contained 14 genes. While these genes had similarities in patterns of expression across a group of 15 tissues, their most prominent shared characteristic was preferential expression in thymus. They were diverse in function. For instance, the group comprised transcription factors *Ets1 *and *Tcf12*, chromatin matrix associated protein *Smarcf1 *(recently renamed *Arid1a*), the ATP-binding cassette transporter *Abcg1 *(transports peptides during antigen processing), the 2'-5'-oligoadenylate synthetase *Oasl2 *that is induced by interferon, and the histocompatibility antigen *H2-K *that plays a role in antigen presentation and processing. Sequences of both the mouse gene and its human ortholog were available for seven genes (*Abcg1*, *Ctsl*, *Man2b1*, *Sgpl1*, *Arid1a*, *Tcf12*, and *Zfp162*). The 3 kb upstream regions of all 7 genes were compared to identify modules of shared *cis*-elements. The search criteria were limited by (1) requiring modules to contain at least 3 TF binding sites, one of which is a lymphoid element (see this list of lymphoid elements in Methods), (2) to be evolutionarily conserved, that is, to occur within the phylogenetic footprints in the aligned mouse-human orthologs, and (3) to be located within 3 kb upstream and 100 bp downstream of the first bp of exon 1 (transcription start). Examples of modules of *cis*-elements are shown in Table 2. *Arid1a*, *Abcg1 *and *Sgpl1 *are most similar to one another. They also have the most similar patterns of expression across tissues, when clustered in hierarchical trees. One module, AP2F EGRF MAZF SP1F ZBPF, contains 5 *cis*-elements within a 200 bp window and is present within 3 kb upstream of transcription start in *Arid1a*, *Abcg1 and Sgpl1*. The conserved modules containing multiple transcription factor binding sites (Table 2 and Figures 3 and 4; Additional file 11 gives fasta sequences, list of binding sites and coordinates) are likely to play a role in regulation of expression of these genes, but this hypothesis must be experimentally verified. *Ctsl*, *Man2b1*, *Zfp162 and Tcf12 *did not share modules (within upstream 3 kb region and having at least one "lymphoid element") with the other genes.

**Table 2:** Examples of modules of shared *cis*-elements in K-cluster, set 15 genes. All elements of a module are within a 200 bp window and are present in both the human and mouse orthologs. Modules are located within 3-kb upstream of the transcription start site.

	**Genes**						
	
**Modules of shared *cis*-elements**	***Arid1a***	***Abcg1***	***Sgpl1***	***Man2b1***	***Ctsl***	***Zfp162***	***Tcf12***
AP2F EGRF MAZF SP1F ZBPF	+	+	+	-	-	-	-
AP2F EGRF SP1F ZBPF	+	+	+	-	-	-	-
AP2F MAZF SP1F ZBPF	+	+	+	-	-	-	-
AP2F HESF MAZF SP1F	+	+	+	-	-	-	-
MAZF SP1F ZBPF	+	+	+	-	-	-	-
AP2F MAZF SP1F	+	+	+	-	-	-	-
AP2F SP1F ZBPF	+	+	-	-	-	-	-
EGRF MAZF ZBPF	+	+	+	-	-	-	-
ETSF SP1F ZBPF	+	+	+	-	-	-	-
AP2F EGRF ETSF SP1F ZBPF	+	+	+	-	-	-	-
AP2F MAZF MZF1 SP1F ZBPF	+	+	-	-	-	-	-
AP2F MAZF MZF1 SP1F	+	+	-	-	-	-	-
AP2F MZF1 SP1F ZBPF	+	+	-	-	-	-	-
ETSF MAZF SP1F	+	-	+	-	-	-	-
EGRF ETSF ZBPF	+	-	+	-	-	-	-

Figure 4 shows the computationally predicted arrangement of *cis*-elements immediately upstream of the transcription start site (promoters) of specific individual genes: *Arid1a*, *Abcg1*, and *Zfp162*. Elements were required to be within 500 bp of transcription start to be shown in Figure 4, which focuses on sequence conservation in classical promoters of pairs of orthologs and does not require that elements be shared with other genes. Modules in Table 2 were within 3 kb of transcription start, which could include both classical promoters and upstream enhancers, and were shared by more than one pair of orthologs. A number of the elements of modules listed in Table 2 are also present in the predicted promoters. For example, MAZF and SP1F are also present in the promoters of *Arid1a*, *Abcg1 *and *Zfp162*.

#### K-cluster Set 7

K-Cluster set 7 includes 19 genes. As a group the genes were better expressed in stimulated lymph nodes and activated T-cells than in the other tissues. Expression was characteristically low in peripheral blood mononuclear cells and in other non-immune adult and fetal tissues. Among the genes in set 7 are the integral surface membrane protein *CD72 *found on B-cells, the transcription regulators I*rf5 *and *Icsbp1*, the tyrosine kinases *Hck*, *Stk10*, and *Lyn *that are a part of the intracellular signaling cascade, the mitogen activated protein kinases *Map3k1 *and *Map4k1 *that participate in the very earliest steps of induction of new gene expression after lymphocytes are exposed to antigen, and the ATP-binding cassette transporters *Abca7 *and *Tap1 *of the type that transport peptides during antigen processing. Other less well-characterized genes in these sets may have functions similar to the genes that are better annotated. Sequences of 11 genes from Set 7 and their human orthologs were examined for the presence of clusters of TF binding sites, at least one of which is a lymphoid element, as defined in Methods. The 11 genes shared relatively few clusters of TF binding sites. There were 7 clusters shared by 3 genes. The largest cluster contained 6 elements, AP2F CDEF EGRF SP1F ZBPF ZF5F and was shared by *Irf5 *and *Stk10*. There were 21 clusters shared by 2 genes and containing 3 to 6 TF binding sites. The composition and location of these are shown in images from CisMols (Additional files 3,4,5,6,7,8 and 9).

#### Highly expressed genes

In addition to searching for potential regulatory regions within sets of genes clustered by similarities of patterns of expression across sets of tissues and within regions immediately upstream of exon 1, we also sought to identify genes characterized by high expression in specific immune tissues. It is not known whether clustering by pattern of expression across tissues and/or grouping by high expression in specific tissues (or neither) will be a useful way to group genes for computational identification of regulatory elements and regulatory regions. It is clear, however, that although modules of *cis-*elements that regulate expression of genes in tissues can occur at many different locations relative to a gene's promoter, at least some regulatory elements are located within promoter regions and this is the region we have searched most intensively for conservation of known TF binding sites. For the purposes of this analysis, we defined genes that were highly expressed based on their normalized expression being at least 4 times higher in an individual immune tissue relative to their median signal across the entire database. High expression in a single tissue does not preclude significant expression in other tissues, so high expression is not synonymous with unique expression. We examined highly expressed mouse genes and their human orthologs for the presence of clusters of TF binding sites, with the additional constraint that at least one of the cis elements present in the cluster was a lymphoid element, as defined in Methods. Grouped by tissue, suitable paired mouse/human orthologs were: activated T-cells, 17 genes: *Ctsz*, *Kpnb1*, *Tnfrsf9*, *Tnfrsf4*, *Myc*, *Mcm2*, *Mcm5*, *Mcm6*, *Mcm7*, *Gzmb*, *Ncf4*, *Gapd*, *Ccl4*, *Pcna*, *Rpl13*, *Cd86*, *Icsbp1*; thymus, 7 genes: *Satb1*, *Hdac7a*, *Sgpl1*, *Abca1*, *Prss16*, *Abcg1*, *C1qg*; stimulated lymph node, 4 genes, *Stk10*, *Irf5*, *Cxcl9*, *Tnfrsf1*. Identical analyses of 6 genes highly expressed in skeletal muscle (*Ckm*, *Myf6*, *Aldo1*, *Myog*, *Dmd*, *Chrm3*) and 8 in liver (*G6pc*, *Cyp7a1*, *Proc*, *Ttr*, *Aldo2*, *Ins2*, *Igf1*, *Pah*) served as negative controls, i.e. not tissues that play a critical role in lymphocyte differentiation or the immune response. The MCM family and *Myc *are involved in replication of DNA and chromosomes. The TNF and TNFR families of genes encode receptors and ligands that couple directly to signaling pathways for cell proliferation, survival and differentiation [41]. *Prss16 *encodes a thymus specific protease which is specifically expressed by epithelial cells in the thymic cortex and plays a role in T-cell development and, perhaps, in susceptibility to autoimmunity [42]. *Hdac7a *encodes a histone deacetylase. Members of the *Hdac *family of genes modify histones and play a role in the regulation of expression of genes such as those functioning in the cell cycle, apoptosis, and transcription [43]. Cxcl9 is an inflammatory chemokine induced by interferon. Its promoter contains binding sites for CREB, STAT1, and NFKB [44].

The results of the above approach are shown in Table 3, which lists examples of putative computationally identified regulatory modules of immune genes and the *cis*-elements that they contain. When modules of genes highly expressed in thymus, stimulated lymph nodes, or activated T-cells were compared with one another and to modules of genes expressed in muscle and liver, it is clear that the composition (*cis*-elements) of modules are not unique to a specific gene. However there is some evidence of unique arrangements of elements within modules. There are also *cis*-elements that are not commonly shared. For example, the individual *cis*-elements HESF, HAML, MYT1 and P53F were not found in modules of genes other than those highly expressed in thymus. Likewise, E2FF was only present in modules of activated T-cells, but clearly does not play a unique role in the immune system. Members of the E2F family of TFs are key participants in cell proliferation, apoptosis, and differentiation [45]. E2FF is found in promoters of *Mcm2*, *Mcm5*, *Mcm6*, *Mcm7*, and *Myc*. These genes are highly expressed in proliferating cells generally, an example of which is the activated T-cell.

**Table 3:** Examples of modules of shared cis-elements found in highly expressed genes in 3 immune tissues. Elements were clustered with at least 2 other cis-elements within a 200 bp window, indicating the presence of a putative regulatory module which contained at least 3 transcription factor binding sites, one of which was required to be a lymphoid element. All are located within 3 kb upstream and 100 bp downstream of the first bp of exon 1. The modules were present in the mouse and human orthologs of at least 2 genes from sets of genes that were highly expressed in thymus, stimulated lymph nodes, or activated T-cells. The number of genes for which orthologs were available: thymus, 7; lymph node, 4; activated T-cells, 17.

**Thymus**	**Stimulated Lymph Node**	**Activated T-cell**
MAZF SP1F ZBPF	AP2F CDEF EGRF SP1F ZBPF ZF5F	MAZF SP1F ZBPF
EGRF MAZF SP1F ZBPF	LHXF NKXH OCT1 RBIT	CREB SP1F ZBPF
ETSF SP1F ZBPF	EGRF ETSF NFKB	E2FF MAZF SP1F
AP2F EGRF HESF MAZF SP1F ZBPF	GATA HOXF NKXH	ECAT PCAT SP1F ZBPF
ETSF MAZF SP1F STAT ZBPF	LHXF NKXH OCT1	ETSF MAZF MZF1
AP2F EGRF ETSF SP1F ZBPF	NKXH OCT1 RBIT	EGRF SP1F ZBPF
EGRF MAZF P53F SP1F		IKRS MAZF NFKB
GATA HAML MYT1		E2FF EBOX ETSF MAZF SP1F ZF5F
		BCL6 CREB E2FF STAT
		HOXF LEFF LHXF OCT1
		MAZF MZF1 NFKB PAX5

Regulatory modules, which have been proved biologically to regulate expression of genes, contain multiple TF binding sites, much as is shown in Figures 2, 3 and 4. Examples of modules of shared *cis*-elements (i.e., within a 200 bp window) in highly expressed genes are listed in Table 3. For example, of the modules highly expressed in thymus SP1F MAZF ZBPF was present in paired orthologs of *Abca1*, *C1qg*, *Abcg1 *and *Sgpl1*; module AP2F EGRF HESF MAZF SP1F ZBPF was present in *Sgpl1 *and *Abcg1*. Of the modules highly expressed in stimulated lymph nodes, AP2F CDEF EGRF SP1F ZBPF ZF5F was present in *Stk10 *and *Irf5*; module GATA HOXF NKXH was present in *Stk10 *and *Irf5*. Of the modules highly expressed in activated T-cells, E2FF EBOX ETSF MAZF SP1F ZF5F was present in *Kpnb1 *and *Mcm6*; module BCL6 CREB E2FF STAT was present in *Icsbp1 *and *Tnfrsf4*.

## Discussion

Individual differentiated biological states can be characterized by gene expression profiling. Large-scale comparisons of profiles of cells, tissues, and developmental stages have the potential to identify a wealth of coordinately regulated groups of genes that reflect the interplay of their functional relationships and transcriptional control mechanisms. We have built a database comprised of the mRNA expression profiles of 65 normal adult and fetal C57BL/6J mouse tissues using the Incyte Mouse GEM1, 8638 element, clone set. Using microarray analysis, 680 sequences were identified that were highly expressed in one or more of 6 immune tissues. Many were also expressed in certain other tissues. Some of these other tissues were organs such as heart, kidney, and brain which do not normally contain lymphocytes in large numbers and do not play a role in the immune response. Others, such as intestine and lung, interface with the external environment, contain significant numbers of lymphocytes, and can mount an immune response. The 680 expressed sequences were filtered to remove 320 that were expressed in "non-immune" brain or heart or kidney. This resulted in a list of 360 expressed sequences called "immune genes" that were less broadly expressed in tissues without immune function than were the 680. Mutations and polymorphisms in both the 680 expressed sequences and the 360 immune genes have a significant chance of specifically affecting immune function. We predict this will be more common with changes in the 360 immune genes. We tested this by comparing reports of disease causing mutations in the 360 immune genes with those reported for the 320 genes that were more broadly expressed (Online Mendelian Inheritance in Man). Of the 360 mouse immune genes, 32 had an ortholog with gene symbol in OMIM and17 had annotations that described a function clearly linked to development or function of the immune system. Mutations in 2 (*LCP2 *and *PARVG*) cause severe immunodeficiency disease. Examples of other diseases caused by mutations in these 32 genes were B- and T-cell malignancies, autoimmune disorders, and reduced viral or bacterial resistance. Of the 320 genes removed from the list of 680, 37 had orthologs with gene symbols listed in OMIM. 4 genes were expressed in lymphocytes and mutation in one, Bruton's tyrosine kinase, causes agammaglobulinemia. Mutations in other genes caused disorders of coagulation, red cells, or granulocytes, rather than the immune system. We conclude that the list of 360 immune genes includes a higher percentage of genes preferentially expressed in immunocompetent tissues and with more specific immune-related functions than does the full list of 680 sequences expressed in immune tissues, but also with expression in non-immune tissues.

The 360 immune genes represent a portion of the complete set of genes that encode proteins and processes necessary for the differentiation, maintenance, and function of the immune system. These genes are functionally diverse and represent both ubiquitous and specialized cellular processes. 10 or more genes are in specific functional clusters that carry out general processes such as DNA and chromosomal replication, cell cycle regulation, transcription, and translation. Other genes are in functional clusters that carry out specialized functions, largely restricted to immune tissues. These include genes that encode proteins involved in antigen recognition and transport, chemokine synthesis, chemokine recognition, and the intracellular signaling cascade necessary to initiate transcription and new protein synthesis in lymphocytes, as part of the host response to antigen. Functional annotation of these genes is a work in progress. While probable functions have been assigned to most of the expressed sequences and the genes that encode them, using information shown in the Additional file 1, there is much work to be done. Most functional annotations are based on the sharing of presently known protein domains and sequence homologies and provide general clues to the role a gene or protein may play in cells that participate in the immune response to antigen. A more precise understanding will come about as new laboratory data are correlated with studies of the expression of specific immune genes, their coordination with expression of other genes, and the structure and function of their products.

For several reasons, the "immune genes" that we have identified are not all of the genes that are expressed in immune tissues: (1) the Incyte set of 8638 genes probably contains representative cDNAs from 25% or less of all mouse genes; (2) genes that are essential to immune function, but are expressed at similar levels in immune and other tissues will not be included in the immune set; and (3) a gene with a very low level of expression will be missed, if cDNA made from its RNA is not present in sufficient quantity to give a signal on the microarray. Genes may be included or excluded in error because of the large number of genes screened for expression with a limited number of replicates. Incyte cDNA microarrays are no longer manufactured and no Incyte arrays or public databases are available to check expression of our immune genes in other species. There are two relevant publicly available Novartis gene expression databases (Genomics Institute of the Novartis Research Foundation, [18]), which can be accessed. One uses Affymetrix chip U74Av2 and a set of 90 mouse tissues and cell lines and the second uses Affymetrix HG U133 and 158 human tissues and cells. Relating Affymetrix probes to Incyte cDNA probes is complex and the Novartis tissue sets do not contain the same tissues we have used. However, our immune genes, when expressed on the Novartis arrays, are generally clustered in tissues of the immunohematopoietic system, the gastrointestinal tract, and lung. These types of publicly available databases will permit identification and functional annotation of new immune genes with consequent availability of larger sets of coordinately regulated genes for searches of conserved regulatory modules.

Using comparative genomics-based, *cis*-element analyses ([15] and [46]), we identified compositionally similar clusters of *cis*-elements in upstream regions of mouse/human orthologs of several immune genes. There was an excellent agreement between the computationally predicted and experimentally determined arrangements of *cis*-elements in the promoters of the mouse *H2-K *and human *HLA-A *genes. Analyses of other immune genes identified a wealth of potential immune system-specific regulatory modules. For example (Table 2), *Arid1a*, *Abcg1*, and *Sgpl1 *are members of a K-clustered set of immune genes and share a phylogenetically conserved module of 5 *cis*-elements: AP2F EGRF MAZF SP1F and ZBPF, all within a 200 bp interval. Other examples of clustered TF binding sites that could be within regulatory modules of genes highly expressed in specific tissues are given in RESULTS. Striking examples of putative modules include the 6 *cis*-element module AP2F EGRF HESF MAZF SP1F ZBPF in genes highly expressed in thymus; the 6 *cis*-element module E2FF EBOX ETSF MAZF SP1F ZFSF in genes highly expressed in activated T-cells; and the 6 element module AP2F CDEF EGRF SP1F ZBPF ZF5F in genes highly expressed in stimulated lymph nodes (Table 3). Putative regulatory modules are not distributed randomly across an entire segment of DNA, but are highly clustered within distinct short segments that are the computationally identified promoters and enhancers (Figure 3). Because of the nature of the scanning algorithm with its 200 bp window, variations of multiple modules may occur within one segment. These phenomena are more easily understood by examining Figure 3. Our data support the hypothesis that (1) regulatory modules of genes are highly clustered in a few sites that can be computationally identified, (2) modules in different genes may share *cis*-elements that bind TFs, and (3) certain combinations of TF binding sites are phylogenetically conserved and appear to be reused across genes when specific patterns of expression are required. *Cis*-elements from the same family have a high probability of interacting with similar groups of transcription factors, although they will not necessarily be in the same position relative to the transcription start site. We have identified genes and putative regulatory modules that play a role in the differentiation, maintenance, and function of the immune system. These results serve to advance both our understanding of normal gene and immune system function and also to identify genes and regulatory regions whose mutation or polymorphic variation lead to immunologic disease.

## Methods

C57BL/6J mice from The Jackson Laboratory were the source of normal adult and fetal tissues. The complete panel of tissues for microarray analyses by our group has been described [47]. Peripheral blood mononuclear cells were separated from whole blood on Ficoll/Hypaque gradients; unstimulated lymph nodes, spleen, and thymus were each collected from unimmunized mice and pooled separately; "stimulated" lymph nodes were collected from mice 10 days after they were immunized with hen egg-white lysozyme (HEL) in complete Freund's adjuvant; activated T cells were prepared by enriching T cells from peripheral blood and treating them with anti-CD3 and anti-CD28. Except for activated T-cells and pancreatic islet cells, all cells and tissues were collected in duplicate. 128 preparations of poly (A)-RNA were made from 65 different tissues, checked for quality, and quantified as previously described [19,47].

Microarray analyses were carried out using Incyte mouse GEM1 cDNA arrays (Incyte Genomics, Palo Alto, CA), as described previously for our group [19,47]. Relative abundance of probes was calculated as the ratio of the sample value against the value from the labeled whole mouse reference cDNA for each gene on each array. Data analyses were carried out with GeneSpring version 4.2.1 (Silicon Genetics) software, including filtering, K-means and hierarchical clustering. A list of all tissues in the full set of 65 normal adult and fetal tissues is provided in Additional file 12. Our analyses focused on comparison gene expression in 18 tissues that were selected to represent a variety of adult and fetal tissues (Figure 1B), most with immunological function. 6 of the 18 tissues were the "immune tissues" – unstimulated and stimulated lymph nodes, spleen, peripheral blood mononuclear cells, activated T-cells, and thymus. The remaining 12 tissues of the 18 tissue set were: fetal day 16.5 intestine and lung; adult duodenum, jejunum, ileum, proximal and distal colon; adult lung and liver, and joint synovium from normal adult mice and mice with acute and chronic arthritis. All pertinent microarray data are available through the Children's Hospital Research Foundation expression database web server  within the ExpressionDB folders of the Incyte Mouse GEM1 chip genome.

Genes on the Incyte array were identified by NCBI GenBank accession and systematic numbers and by gene symbol, where available. For those sequences that could not be assigned a gene symbol, sequence homologies to known mouse genes were sought using MouseBLAST [23], BLAT [12], MGD [23], and LocusLink [24]. BLAST comparisons of the human and mouse confirmed Ensembl predictions of human orthologs of mouse genes. Identity of genes was confirmed by BLAST comparison of the GenBank sequences from NCBI [48] with Ensembl [13] sequences. When downloading the genomic sequences with flanking sequences, it was important to have an mRNA that contained exon 1, so the site of initiation of transcription was correctly identified. Presence of an upstream exon 1 in an isoform would lead to re-defining of the promoter and intronic regions. Criteria for presence of exon 1 included: comparison of the number and location of exons in orthologous genes, alignment of transcripts of the gene as reported by different databases, and alignment of the 5' end of the transcript with the putative start site and signals in the gene. In cases where we encountered multiple high scoring transcript hits against the genome, we manually looked into the alignments to rule out the occurrence of pseudogenes that frequently lacked introns when compared to the "true" genes. Additional information about sequences of both the transcript and the gene was obtained from UCSC Golden Path [12]. Confirmation of the presence of exon 1 in orthologs was particularly important because of the need to locate the start site of transcription. Computational prediction of exons is error prone. DNA sequences of genes were downloaded to include at least 10,000 flanking base pairs upstream and downstream of the first and last exons respectively. The November 2002 and April 2003 assemblies of human and the February 2002 and February 2003 assemblies of mouse genome were used for this purpose depending upon their availability at the time of our analyses (Additional files 10 and 11 list relevant FASTA sequences and genomic coordinates).

The GO and MGI databases were searched for annotations of the immune genes, using Stanford SOURCE [40]. For genes not found or incompletely annotated, manual annotation was done using criteria similar to the Gene Ontology (GO) [49], Mouse Genome Informatics (MGI) [23], and LocusLink classifications [50]. A function was assigned if the encoded protein contained distinctive InterPro functional domains, or sequence similarity to paralogs previously annotated, or sequence similarity to functionally characterized SwissProt/TrEMBL proteins. Using the information about structure and function, the authors simplified annotations and grouped genes by major functions, such as antigen binding and processing (defense – immune function), transcription, protein synthesis, apoptosis, cell division. Highly detailed annotations are provided in the supplementary materials (Additional file 1).

To identify putative consensus *cis*-acting regulatory sequences in genes that were coordinately regulated, we first selected groups of genes based on their expression patterns in different immune tissues. The complete genomic sequences (with flanking upstream and downstream regions of 40 kb) of the selected genes and their orthologs were extracted from the Ensembl/UCSC human and mouse databases [12,13]. Where available the NCBI-RefSeq mRNAs were used as references for downloading the genomic sequences with upstream and downstream gene flanking regions of 40 kb. The transcription start site was thus at 40,000 in the downloaded sequences used in comparative genomic analysis for identification of potential regulatory clusters using Trafac server [15]. Repeat elements were masked using the RepeatMasker [51]. Conserved clusters of regulatory elements in the evolutionarily conserved non-coding regions of mouse and human orthologs were displayed using the TraFaC [15] or GenomeTraFaC [46] servers which integrate results from MatInspector Professional (Version 4.1, 2004; 356 individual matrices in 138 families) [52] and Advanced PipMaker (chaining option) [53] programs. We compared conserved putative *cis*-regulatory regions of each of the different groups of genes from mouse and human to identify known TF binding sites. The CisMols analyzer [22] permits selection of TFs that must be present in clusters of TFs that constitute a putative regulatory module. To convey specificity to the search for modules relevant to regulation of gene expression in immune tissues, we required the presence of one or more of the following TFs, which we call "lymphoid elements". They have been reported to play a role in some aspect of lymphoid biology (see for example, [1-3]: BCL6, CMYB, CREB, EGRF, ETSF, GATA, IKRS, IRFF, MZF1, NFAT, NFKB, OCT1 (site also binds OCT2), PAX5, SP1F, VMYB, and WHZF. ECAT and PCAT were also included because of their frequent occurrence in promoters at the start of transcription. The search was limited to a region 3 kb upstream and 100 bp downstream of the start site of exon 1 (based on the NCBI-RefSeq mRNA annotations). This is where the promoter and associated regulatory elements would be expected, given that additional regulatory elements (enhancers/silencers) are almost certain to be located elsewhere. Images of the CisMols analyses of genes to identify regulatory elements are also provided in supplementary materials (Additional files 3 to 9). One example is shown in Figure 3.

## Authors' contributions

JJH and BJA were primarily responsible for the design, coordination and conduct of the study. AGJ and AG were responsible for regulatory region analyses and software development. AGJ was responsible for ortholog analysis and novel ortholog assignments. JJH, BJA and AGJ drafted the manuscript and figures and JJH, BJA and AGJ contributed editorial revisions. SK, SW and CE were responsible for generating, quality assurance, and initial assembly of the gene chip data. JDK provided purified lymphoid cells, read the manuscript and provided comments and discussion. All authors read and approved the final manuscript.

## Supplementary Material

Additional File 1 List and annotation of 360 expressed sequences ("immune genes"). Click here for file

Additional File 10 FASTA sequences and the corresponding coordinates on the human and mouse genome assemblies (May 2004) of the promoter regions used in the analysis and displayed in figures 2 and 4. Click here for file

Additional File 2 Using K-means clustering in GeneSpring (Version 4.2.1), 160 annotated genes, were divided into distinct sets based on similarity of expression patterns across 15 tissues. Tissues were given equal weight, the number of clusters was set at 20, and similarity was measured by standard correlation. Click here for file

Additional File 11 FASTA sequences and the corresponding coordinates on the human and mouse genome assemblies (May 2004) of the promoter regions used in the analysis and displayed in figures 2 and 4. Click here for file

Additional File 3 CisMols display of location and composition of clusters of cis-elements that are putative regulatory modules for the genes in various groups (test and control). Each colored cube indicates a cluster of 3 or more cis-elements with at least one "lymphoid element". The region searched is upstream 3 kb and downstream 100 bp of transcription start site (as defined by the respective mRNAs from NCBI's RefSeq database). The legend in the lower left half of the figure indicates the composition of each of the modules and the genes that share them. Click here for file

Additional File 4 CisMols display of location and composition of clusters of cis-elements that are putative regulatory modules for the genes in various groups (test and control). Each colored cube indicates a cluster of 3 or more cis-elements with at least one "lymphoid element". The region searched is upstream 3 kb and downstream 100 bp of transcription start site (as defined by the respective mRNAs from NCBI's RefSeq database). The legend in the lower left half of the figure indicates the composition of each of the modules and the genes that share them. Click here for file

Additional File 5 CisMols display of location and composition of clusters of cis-elements that are putative regulatory modules for the genes in various groups (test and control). Each colored cube indicates a cluster of 3 or more cis-elements with at least one "lymphoid element". The region searched is upstream 3 kb and downstream 100 bp of transcription start site (as defined by the respective mRNAs from NCBI's RefSeq database). The legend in the lower left half of the figure indicates the composition of each of the modules and the genes that share them. Click here for file

Additional File 6 CisMols display of location and composition of clusters of cis-elements that are putative regulatory modules for the genes in various groups (test and control). Each colored cube indicates a cluster of 3 or more cis-elements with at least one "lymphoid element". The region searched is upstream 3 kb and downstream 100 bp of transcription start site (as defined by the respective mRNAs from NCBI's RefSeq database). The legend in the lower left half of the figure indicates the composition of each of the modules and the genes that share them. Click here for file

Additional File 7 CisMols display of location and composition of clusters of cis-elements that are putative regulatory modules for the genes in various groups (test and control). Each colored cube indicates a cluster of 3 or more cis-elements with at least one "lymphoid element". The region searched is upstream 3 kb and downstream 100 bp of transcription start site (as defined by the respective mRNAs from NCBI's RefSeq database). The legend in the lower left half of the figure indicates the composition of each of the modules and the genes that share them. Click here for file

Additional File 8 CisMols display of location and composition of clusters of cis-elements that are putative regulatory modules for the genes in various groups (test and control). Each colored cube indicates a cluster of 3 or more cis-elements with at least one "lymphoid element". The region searched is upstream 3 kb and downstream 100 bp of transcription start site (as defined by the respective mRNAs from NCBI's RefSeq database). The legend in the lower left half of the figure indicates the composition of each of the modules and the genes that share them. Click here for file

Additional File 9 CisMols display of location and composition of clusters of cis-elements that are putative regulatory modules for the genes in various groups (test and control). Each colored cube indicates a cluster of 3 or more cis-elements with at least one "lymphoid element". The region searched is upstream 3 kb and downstream 100 bp of transcription start site (as defined by the respective mRNAs from NCBI's RefSeq database). The legend in the lower left half of the figure indicates the composition of each of the modules and the genes that share them. Click here for file

Additional File 12 Tissue lists used in the generation of microarray profile data. Click here for file
