# Supplementary material for: Microarray and comparative genomics-based identification of genes and gene regulatory regions of the mouse immune system
Source: BMC Genomics. 2004 Oct 25;5:82. doi: 10.1186/1471-2164-5-82 (PMC534115; doi:10.1186/1471-2164-5-82)

**Fasta sequences of human HLA-A and mouse H2-K**:

>hgNM_002116 HLA-A Human Promoter region (extracted from 9696 to 10098) Correspond to chr6:30,015,866-30,016,268 (+) of the Human Genome July 2003 Assembly (Relative positions to ATG: -305 to +97)

TCGCACAGGAGCAGAGGGGTCAGGGCGAAGTCCCAGGGCCCCAGGCGTGGCTCTCAGAGTCTCAGGCCCC

GAAGGCGGTGTATGGATTGGGGAGTCCCAGCCTTGGGGATTCCCCAACTCCGCAGTTTCTTTTCTCCCTC

TCCCAACCTACGTAGGGTCCTTCATCCTGGATACTCACGACGCGGACCCAGTTCTCACTCCCATTGGGTG

TCGGGTTTCCAGAGAAGCCAATCAGTGTCGTCGCGGTCGCTGTTCTAAAGCCCGCACGCACCCACCGGGA

CTCAGATTCTCCCCAGACGCCGAGGATGGCCGTCATGGCGCCCCGAACCCTCCTCCTGCTACTCTCGGGG

GCCCTGGCCCTGACCCAGACCTGGGCGGGTGAGTGCGGGGTCGGGAGGGAAAC

>mgXM_193866 H2-K Mouse Promoter region (extracted from 9810 to 10170) Correspond to chr17:33,638,839-33,639,199 (-) of the Mouse October 2003 Assembly (Relative positions to ATG: -653 to -293)

GCACAGGGTTCAGGCAAAGTCTTAGTCGCCAGGCAGTGAGGTCAGGGGTGGGGAAGCCCAGGGCTGGGGA

TTCCCCATCTCCACAGTTTCACTTCTGCACCTAACCTGGGTCAGGTCCTTCTGTCCGGACACTGTTGACG

CGCAGTCAGCTCTTACCCCCATTGGGTGGCGCGATCACCCAAGAACCAATCAGTGTCGCCGCGGACGCTG

GATATAAAGTCCACGCAGCCCGCAGAACTCAGAAGTCGCGAATCGCCGACAGGTGCGATGGTACCGTGCA

CGCTGCTCCTGCTGTTGGCGGCCGCCCTGGCTCCGACTCAGACCCGCGCGGGTGAGTACCGGGCCGGGAG

GGAAACGGCCT

**Alignment of above sequences:**

Sequence 1 lcl|hgNM_002116 HLA-A Human Promoter region (extracted from 9696 to 10098) Correspond to chr6:30,015,866-30,016,268 (+) of the Human Genome July 2003 Assembly (Relative positions to ATG: -305 to +97) Length 403 (1 .. 403)

Sequence 2 lcl|mgXM_193866 H2-K Mouse Promoter region (extracted from 9810 to 10170) Correspond to chr17:33,638,839-33,639,199 (-) of the Mouse October 2003 Assembly (Relative positions to ATG: -653 to -293) Length 361 (1 .. 361)

Score = 56.4 bits (29), Expect = 4e-05Identities = 45/53 (84%) Strand = Plus / Plus

Human: 351 gccctggccctgacccagacctgggcgggtgagtgcggggtcgggagggaaac 403

|||||||| | ||| |||||| | |||||||||| | ||| ||||||||||||

Mouse: 304 gccctggctccgactcagacccgcgcgggtgagtaccgggccgggagggaaac 356

Score = 48.8 bits (25), Expect = 0.009Identities = 120/165 (72%), Gaps = 3/165 (1%) Strand = Plus / Plus

Human: 88 tggggagtcccagccttggggattccccaactccgcagtttcttttctccctctcccaac 147

|||||| ||||| ||||||||||||| |||| ||||||| |||| | || |||

Mouse: 49 tggggaagcccagggctggggattccccatctccacagtttcacttct---gcacctaac 105

Human: 148 ctacgtagggtccttcatcctggatactcacgacgcggacccagttctcactcccattgg 207

|| || |||||||| | ||| ||| |||||| | ||| ||| || ||||||||

Mouse: 106 ctgggtcaggtccttctgtccggacactgttgacgcgcagtcagctcttacccccattgg 165

Human: 208 gtgtcgggtttccagagaagccaatcagtgtcgtcgcggtcgctg 252

||| || | | | | | ||||||||||||| ||||| |||||

Mouse: 166 gtggcgcgatcacccaagaaccaatcagtgtcgccgcggacgctg 210

**Alignment in the context of sequences used in Trafac:**

Sequence 1 lcl|hgNM_002116 HLA-A Human Length 403 (9696 .. 10098)

Sequence 2 lcl|mgXM_193866 H2-K Mouse Length 361 (9810 .. 10170)

Score = 56.4 bits (29), Expect = 4e-05Identities = 45/53 (84%) Strand = Plus / Plus

Human: 10046 gccctggccctgacccagacctgggcgggtgagtgcggggtcgggagggaaac 10098

|||||||| | ||| |||||| | |||||||||| | ||| ||||||||||||

Mouse: 10113 gccctggctccgactcagacccgcgcgggtgagtaccgggccgggagggaaac 10165

Score = 48.8 bits (25), Expect = 0.009Identities = 120/165 (72%), Gaps = 3/165 (1%) Strand = Plus / Plus

Human: 9783 tggggagtcccagccttggggattccccaactccgcagtttcttttctccctctcccaac 9842

|||||| ||||| ||||||||||||| |||| ||||||| |||| | || |||

Mouse: 9858 tggggaagcccagggctggggattccccatctccacagtttcacttct---gcacctaac 9914

Human: 9843 ctacgtagggtccttcatcctggatactcacgacgcggacccagttctcactcccattgg 9902

|| || |||||||| | ||| ||| |||||| | ||| ||| || ||||||||

Mouse: 9915 ctgggtcaggtccttctgtccggacactgttgacgcgcagtcagctcttacccccattgg 9974

Human: 9903 gtgtcgggtttccagagaagccaatcagtgtcgtcgcggtcgctg 9947

||| || | | | | | ||||||||||||| ||||| |||||

Mouse: 9975 gtggcgcgatcacccaagaaccaatcagtgtcgccgcggacgctg 10019

**List of binding sites and positions in the human and mouse promoter regions of HLA-A and H2-K respectively:**

| Family | Description | hgNM_002116 | | | mgXM_193866 | | |
| --- | --- | --- | --- | --- | --- | --- | --- |
|  |  | Begin | End | Sequence | Begin | End | Sequence |
| V$NFKB | NF-kappaB | 9717 | 9731 | AGGGCGAAGTCCCAG | 9857 | 9871 | GTGGGGAAGCCCAGG |
| V$NFKB | NF-kappaB (p50) | 9782 | 9796 | TTGGGGAGTCCCAGC | 9873 | 9887 | CTGGGGATTCCCCAT |
| V$NFKB | NF-kappaB (p50) | 9798 | 9812 | TTGGGGATTCCCCAA | 9873 | 9887 | CTGGGGATTCCCCAT |
| V$IKRS | Ikaros 1 | 9801 | 9813 | GGGATTCCCCAAC | 9876 | 9888 | GGGATTCCCCATC |
| V$IRFF | interferon-stimulated response element | 9818 | 9832 | CAGTTTCTTTTCTCC | 9893 | 9907 | CAGTTTCACTTCTGC |
| V$WHZF | winged helix protein, involved in hair keratinization and thymus epithelium differentiation | 9872 | 9882 | ACGACGCGGAC | 10011 | 10021 | CGGACGCTGGA |
| V$ECAT | nuclear factor Y (Y-box binding factor) | 9893 | 9907 | CTCCCATTGGGTGTC | 9965 | 9979 | CCCCCATTGGGTGGC |
| V$EKLF | Erythroid krueppel like factor (EKLF) | 9895 | 9905 | CCCATTGGGTG | 9967 | 9977 | CCCATTGGGTG |
| V$ECAT | nuclear factor Y (Y-box binding factor) | 9918 | 9932 | AGAAGCCAATCAGTG | 9990 | 10004 | AAGAACCAATCAGTG |
| V$PCAT | cellular and viral CCAAT box | 9919 | 9929 | GAAGCCAATCA | 9968 | 9978 | CCATTGGGTGG |
| V$PCAT | cellular and viral CCAAT box | 9919 | 9929 | GAAGCCAATCA | 9991 | 10001 | AGAACCAATCA |
| V$TBPF | Muscle TATA box | 9946 | 9962 | TGTTCTAAAGCCCGCAC | 10019 | 10035 | GGATATAAAGTCCACGC |
| V$AHRR | aryl hydrocarbon receptor / Arnt heterodimers | 9952 | 9974 | AAAGCCCGCACGCACCCACCGGG | 10080 | 10102 | GTACCGTGCACGCTGCTCCTGCT |
| V$EGRF | Wilms Tumor Suppressor | 9952 | 9974 | AAAGCCCGCACGCAC | 10005 | 10019 | TCGCCGCGGACGCTG |
| V$EKLF | Erythroid krueppel like factor (EKLF) | 9964 | 9974 | CACCCACCGGG | 9967 | 9977 | CCCATTGGGTG |
| V$CREB | activating transcription factor | 9996 | 10016 | CGAGGATGGCCGTCATGGCGC | 9839 | 9859 | CAGGCAGTGAGGTCAGGGGTG |
| V$CREB | activating transcription factor | 9996 | 10016 | CGAGGATGGCCGTCATGGCGC | 9939 | 9959 | CACTGTTGACGCGCAGTCAGC |

**Trafac image of human and mouse promoter regions of HLA-A and H2-K:**


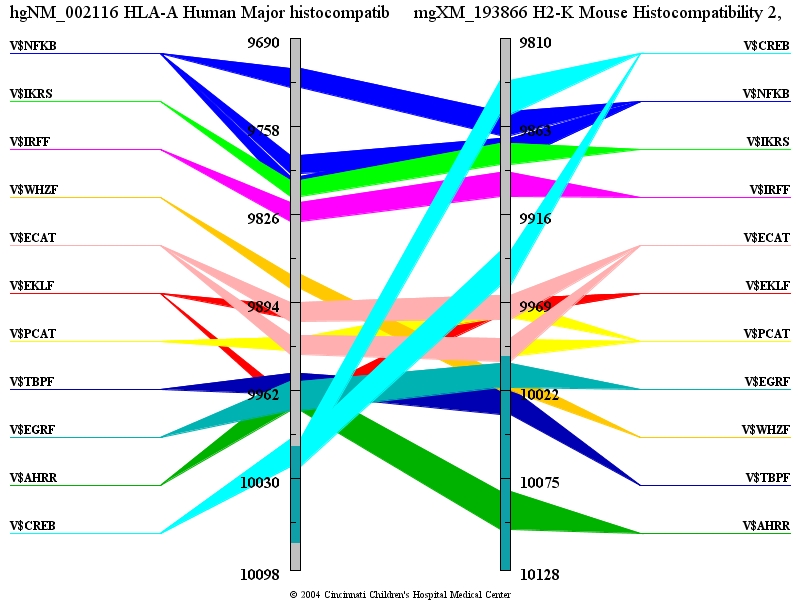

Supplement: Additional File 10 — FASTA sequences and the corresponding coordinates on the human and mouse genome assemblies (May 2004) of the promoter regions used in the analysis and displayed in figures 2 and 4. [file 1471-2164-5-82-S10.doc]
