# Supplementary material for: Microarray and comparative genomics-based identification of genes and gene regulatory regions of the mouse immune system
Source: BMC Genomics. 2004 Oct 25;5:82. doi: 10.1186/1471-2164-5-82 (PMC534115; doi:10.1186/1471-2164-5-82)
Supplement: Additional File 11 — FASTA sequences and the corresponding coordinates on the human and mouse genome assemblies (May 2004) of the promoter regions used in the analysis and displayed in figures 2 and 4. [file 1471-2164-5-82-S11.doc]

**Fasta sequences of the promoter regions of the human genes depicted in Figure 4 of the main text:**

>hgNM_139135 ARID1A human SWI/SNF related, matrix associated, actin dependent regulator of chromatin, subfamily f, member 1 chr1| len=397| comm=extracted from 39924 to 40320, original sequence length: 166071; TSS at 40001

CCCCGCTCCCTCCCTCCCTCCCCGCCCCCTCCCCCTCGCCTCCCTCCCTCCCTCCCTCCTCCTTTCTCCG

GCAGCAGAAAGCGGAGAGTCACAGCGGGGCCAGGCCCTGGGGAGCGGAGCCTCCACCGCCCCCCTCATTC

CCAGGCAAGGGCTTGGGGGGAATGAGCCGGGAGAGCCGGGTCCCGAGCCTACAGAGCCGGGAGCAGCTGA

GCCGCCGGCGCCTCGGCCGCCGCCGCCGCCTCCTCCTCCTCCGCCGCCGCCAGCCCGGAGCCTGAGCCGG

CGGGGCGGGGGGGAGAGGAGCGAGCGCAGCGCAGCAGCGGAGCCCCGCGAGGCCCGCCCGGGCGGGTGGG

GAGGGCAGCCCGGGGGACTGGGCCCCGGGGCGGGGTGGGAGGGGGGG

>hgNM_004915 ABCG1 human ATP-binding cassette, sub-family G| len=205|comm=extracted from 39666 to 39870, original sequence length: 157974; TSS at 40001

GCAGGGGGTTCCCATGCCGCCTGCGAGGCCTCGGCTCGGGCCGCTCCCGGAACCTGCACTTCAGGGGTCC

TGGTCCGCCGCCCCCAGCAGGAGCAAAACAAGAGCACGCGCACCTGCCGGCCCGCCCGCCCCCTTGGTGC

CGGCCAATCGCGCGCTCGGGGCGGGGTCGGGCGCGCTGGAACCAGAGCCGGAGCCGGATCCCAGC

>hgNM_004630 SF1 human splicing factor 1 chr11| len=205|comm=extracted from 39888 to 40092, original sequence length: 94158; TSS at 40001

ATTGATTGAGTCCACCACCGCAGCCAATGAGAGAGCTCGCCGTCGCTCCGTCATAGAGTTCGCCCCACCC

CATCCCCTCCTTTCTGGACTCGGAGCTCAGTTCACGCAGTAACAAATGAAGTGCGCGCTGCGACACCTCC

CAGCCCACCGAACTCCGCCGCCATTTCCTCGCTTGGCCTAACGGTTCGGCCAATCCCAGCGCGCA

**Coordinates of the above sequences on Human Genome Assembly May 2004**

ARID1A: chr1 + 26706590 26706986

ABCG1: chr21 + 42512113 42512317

SF1: chr11 - 64302720 64302924

**Fasta sequences of the promoter regions of the corresponding mouse orthologous genes depicted in Figure 4 of the main text:**

>mgNM_033566 Arid1a mouse SWI/SNF related, matrix associated, actin dependent regulator of chromatin, subfamily f, member 1 chr4| len=397|comm=extracted from 39924 to 40320, original sequence length: 154834; TSS at 40001

NNCCGTTCCCTCCCTCCTTTCCCGCCCCCTCCCCCTCGCCTCCCTCCCTCCCTCCCTCCTCCTTTCTCCG

GCAGCAGAAAGCGGAGAGTCACAGCGGGGCCAGGCCCTGGGGAGCGGAGCCTCCACCGCCCCCCTCATTC

CCAGGCAAGGGCTTGGGGGGAATGAGCCGGGAGAGCCGGGTCCCGAGCCGCCAGAGCCCGGAGCAGCTGA

GCCGCCGGCGCCTCGGCTGCCGCCGCCGCCTCCTCCTCCTCCTCCGCCGCCAGCCCGGAGCCTGAGCCGG

CGGGGCGGGGGGGAGAGGAGCGAGCGCAGCGCAGCAGCGGAGCCCCGCGAGGCCCGCCCGGGCGGGTGGG

GAGGGCAGCCCGGGGGACTCGGCCCCGGGGCGGGGTGGGAGGGGGGA

>mgNM_009593 Abcg1 mouse ATP-binding cassette, sub-family G| len=205|comm=extracted from 39780 to 39984, original sequence length: 138764; TSS at 40001

TCCCTGAAGTGGGCTTCCCAGGCCCAGGCGGTTGGGAGAGGCCTAGCAGACAGCTTCGGGTCCAGAGCAT

CCTCCAGCCGCTCCGCAACAGGAGCAAAACAAGAGCACGCGCACCTGTCGGCGCCCCGCCCCCTCCGCCC

GGCCCAATCGTGGGCAGGGGCGGGGCCGGGGCGCAGTCGGAACCCGCGCAGAGCGCCGCGGAGGA

>mgNM_011750 Zfp162 mouse splicing factor 1 chr19| len=205|comm=extracted from 39504 to 39708, original sequence length: 90811; TSS at 40001

ACGCCGAGGCGCTTACCTCCATCGGTTTCAGCCAATGAGCTAGCCGTTCTCTCACGTCACAGACTTTGCC

CCCTCCTATTCCCCTGCCACTCTGGCCTCAGTCCGCGCAGTAACAAGGAGTGCGCGCTGCGACACCTCCC

AGTTCCCCCGGCCCCGCCGCCATTTCGTCGCCGGGCCTAACGGTTCGGCCAATCCCGACCCATAT

**Coordinates of the above sequences on Mouse Assembly May 2004**

Arid1a: chr4 - 132206952 132207348

Abcg1: chr19 + 6151958 6152162

Zfp162: chr17 + 29663342 29663546

The binding sites - their position, sequence and the definitions for each of the above sequences - can be obtained from http://genometrafac.cchmc.org using either the gene symbols or RefSeq accession numbers for querying. Below is the list of cis-elements, their positions and the sequences for each of the above 3 gene pairs.

***ARID1A*: Human Vs Mouse**

| **Family** | **Description** | **hgNM_139135 (Human ARID1A)** | | | **mgNM_033566 (Mouse Arid1a)** | | |
| --- | --- | --- | --- | --- | --- | --- | --- |
|  |  | **Begin** | **End** | **Sequence** | **Begin** | **End** | **Sequence** |
| V$MAZF | Myc associated zinc finger protein (MAZ) | 39935 | 39952 | CCCTCCCTCCCCG | 39946 | 39958 | CGCCCCCTCCCCC |
| V$ZBPF | Zinc finger transcription factor ZBP-89 | 39938 | 39960 | TCCCTCCCCGCCCCCTCCCCCTC | 39938 | 39960 | TCCTTTCCCGCCCCCTCCCCCTC |
| V$SP1F | stimulating protein 1 SP1, ubiquitous zinc finger transcription factor | 39940 | 39954 | CCTCCCCGCCCCCTC | 39940 | 39954 | CTTTCCCGCCCCCTC |
| V$MZF1 | MZF1 | 39942 | 39948 | TCCCCGC | 40078 | 40084 | GGGGGGA |
| V$ZBPF | Zinc finger transcription factor ZBP-89 | 39944 | 39966 | CCCGCCCCCTCCCCCTCGCCTCC | 39944 | 39966 | CCCGCCCCCTCCCCCTCGCCTCC |
| V$MAZF | MYC-associated zinc finger protein related transcription factor | 39946 | 39958 | CGCCCCCTCCCCC | 39952 | 39969 | CTCCCCCTCGCCT |
| V$SP1F | GC box elements | 39946 | 39960 | CGCCCCCTCCCCCTC | 39946 | 39960 | CGCCCCCTCCCCCTC |
| V$MAZF | Myc associated zinc finger protein (MAZ) | 39952 | 39969 | CTCCCCCTCGCCT | 40204 | 40219 | CGGGGCGGGGGGG |
| V$BARB | barbiturate-inducible element | 39978 | 39992 | CCTCCTCCTTTCTCC | 39978 | 39992 | CCTCCTCCTTTCTCC |
| V$PAX5 | B-cell-specific activating protein | 40002 | 40030 | AAGCGGAGAGTCACAGCGGGGCCAGGCCC | 40002 | 40030 | AAGCGGAGAGTCACAGCGGGGCCAGGCCC |
| V$NOLF | olfactory neuron-specific factor | 40020 | 40042 | GGGCCAGGCCCTGGGGAGCGGAG | 40020 | 40042 | GGGCCAGGCCCTGGGGAGCGGAG |
| V$AP2F | Activator protein 2 | 40025 | 40037 | AGGCCCTGGGGAG | 40025 | 40037 | AGGCCCTGGGGAG |
| V$ZBPF | Core promoter-binding protein (CPBP) with 3 Krueppel-type zinc fingers | 40025 | 40047 | AGGCCCTGGGGAGCGGAGCCTCC | 40025 | 40047 | AGGCCCTGGGGAGCGGAGCCTCC |
| V$ZBPF | Zinc finger transcription factor ZBP-89 | 40042 | 40064 | GCCTCCACCGCCCCCCTCATTCC | 40042 | 40064 | GCCTCCACCGCCCCCCTCATTCC |
| V$SP1F | stimulating protein 1 SP1, ubiquitous zinc finger transcription factor | 40044 | 40058 | CTCCACCGCCCCCCT | 40044 | 40058 | CTCCACCGCCCCCCT |
| V$PAX6 | PAX6 paired domain and homeodomain are required for binding to this site | 40055 | 40073 | CCCTCATTCCCAGGCAAGG | 40055 | 40073 | CCCTCATTCCCAGGCAAGG |
| V$TEAF | TEF-1 related muscle factor | 40057 | 40069 | CTCATTCCCAGGC | 40057 | 40069 | CTCATTCCCAGGC |
| V$IKRS | Ikaros 1, potential regulator of lymphocyte differentiation | 40057 | 40069 | CTCATTCCCAGGC | 40057 | 40069 | CTCATTCCCAGGC |
| V$TEAF | TEF-1 related muscle factor | 40077 | 40089 | TGGGGGGAATGAG | 40077 | 40089 | TGGGGGGAATGAG |
| V$MZF1 | MZF1 | 40078 | 40084 | GGGGGGA | 40211 | 40217 | GGGGGGA |
| V$HEN1 | HEN1 | 40119 | 40140 | GCCGGGAGCAGCTGAGCCGCC | 40119 | 40140 | GCCCGGAGCAGCTGAGCCGCC |
| V$AP4R | Activator protein 4 | 40121 | 40137 | CGGGAGCAGCTGAGCCG | 40121 | 40137 | CCGGAGCAGCTGAGCCG |
| V$BEL1 | Bel-1 similar region (defined in Lentivirus LTRs) | 40124 | 40146 | GAGCAGCTGAGCCGCCGGCGCCT | 40124 | 40146 | GAGCAGCTGAGCCGCCGGCGCCT |
| V$EGRF | Egr-1/Krox-24/NGFI-A immediate-early gene product | 40132 | 40146 | GAGCCGCCGGCGCCT | 40132 | 40146 | GAGCCGCCGGCGCCT |
| V$EGRF | Egr-1/Krox-24/NGFI-A immediate-early gene product | 40147 | 40164 | CGGCCGCCGCCGCCG | 40150 | 40164 | CTGCCGCCGCCGCCT |
| V$EGRF | Egr-1/Krox-24/NGFI-A immediate-early gene product | 40171 | 40185 | CCTCCGCCGCCGCCA | 40304 | 40318 | CGGGGTGGGAGGGGG |
| V$MINI | Muscle Initiator Sequence | 40177 | 40195 | CCGCCGCCAGCCCGGAGCC | 40177 | 40195 | CCGCCGCCAGCCCGGAGCC |
| V$MEF3 | MEF3 binding site, present in skeletal muscle-specific transcriptional enhancers | 40189 | 40201 | CGGAGCCTGAGCC | 40189 | 40201 | CGGAGCCTGAGCC |
| V$ZBPF | Core promoter-binding protein (CPBP) with 3 Krueppel-type zinc fingers | 40196 | 40218 | TGAGCCGGCGGGGCGGGGGGGAG | 40196 | 40218 | TGAGCCGGCGGGGCGGGGGGGAG |
| V$AP2F | Activator protein 2 | 40197 | 40209 | GAGCCGGCGGGGC | 40197 | 40209 | GAGCCGGCGGGGC |
| V$SP1F | stimulating protein 1 SP1, ubiquitous zinc finger transcription factor | 40202 | 40216 | GGCGGGGCGGGGGGG | 40202 | 40216 | GGCGGGGCGGGGGGG |
| V$ZBPF | Zinc finger transcription factor ZBP-89 | 40202 | 40226 | GGCGGGGCGGGGGGGAGAGGAGC | 40202 | 40226 | GGCGGGGCGGGGGGGAGAGGAGC |
| V$MAZF | Myc associated zinc finger protein (MAZ) | 40204 | 40219 | CGGGGCGGGGGGG | 40265 | 40282 | GCGGGTGGGGAGG |
| V$MZF1 | MZF1 | 40211 | 40217 | GGGGGGA | 40269 | 40275 | GTGGGGA |
| V$MTF1 | Metal transcription factor 1, MRE | 40220 | 40234 | GGAGCGAGCGCAGCG | 40220 | 40234 | GGAGCGAGCGCAGCG |
| V$SP1F | stimulating protein 1 SP1, ubiquitous zinc finger transcription factor | 40252 | 40266 | GAGGCCCGCCCGGGC | 40252 | 40266 | GAGGCCCGCCCGGGC |
| V$EKLF | Erythroid krueppel like factor (EKLF) | 40260 | 40277 | CCCGGGCGGGTGG | 40260 | 40277 | CCCGGGCGGGTGG |
| V$MAZF | MYC-associated zinc finger protein related transcription factor | 40265 | 40282 | GCGGGTGGGGAGG | 40299 | 40311 | CGGGGCGGGGTGG |
| V$MZF1 | MZF1 | 40269 | 40275 | GTGGGGA | 40314 | 40320 | GGGGGGA |
| V$NOLF | olfactory neuron-specific factor | 40274 | 40296 | GAGGGCAGCCCGGGGGACTGGGC | 40274 | 40296 | GAGGGCAGCCCGGGGGACTCGGC |
| V$AP2F | Activator protein 2 | 40279 | 40291 | CAGCCCGGGGGAC | 40279 | 40291 | CAGCCCGGGGGAC |
| V$ZBPF | Core promoter-binding protein (CPBP) with 3 Krueppel-type zinc fingers | 40291 | 40318 | CTGGGCCCCGGGGCGGGGTGGGA | 40291 | 40318 | CTCGGCCCCGGGGCGGGGTGGGA |
| V$REBV | Epstein-Barr virus transcription factor R | 40292 | 40312 | TGGGCCCCGGGGCGGGGTGGG | 40292 | 40312 | TCGGCCCCGGGGCGGGGTGGG |
| V$AP2F | Activator protein 2 | 40293 | 40306 | GGGCCCCGGGGCG | 40293 | 40306 | CGGCCCCGGGGCG |
| V$SP1F | stimulating protein 1 SP1, ubiquitous zinc finger transcription factor | 40297 | 40311 | CCCGGGGCGGGGTGG | 40297 | 40311 | CCCGGGGCGGGGTGG |
| V$MAZF | MYC-associated zinc finger protein related transcription factor | 40299 | 40311 | CGGGGCGGGGTGG | 40308 | 40320 | GTGGGAGGGGGGA |
| V$RREB | Ras-responsive element binding protein 1 | 40299 | 40313 | CGGGGCGGGGTGGGA | 40299 | 40313 | CGGGGCGGGGTGGGA |
| V$EGRF | Wilms Tumor Suppressor | 40304 | 40318 | CGGGGTGGGAGGGGG | 40304 | 40318 | CGGGGTGGGAGGGGG |
| V$SP1F | GC box elements | 40306 | 40320 | GGGTGGGAGGGGGGG | 40297 | 40311 | CCCGGGGCGGGGTGG |
| V$MAZF | Myc associated zinc finger protein (MAZ) | 40308 | 40320 | GTGGGAGGGGGGG | 40308 | 40320 | GTGGGAGGGGGGA |

***ABCG1*: Human Vs Mouse**

| **Family** | **Description** | **hgNM_004915 (Human ABCG1)** | | | **mgNM_009593 (Mouse Abcg1)** | | |
| --- | --- | --- | --- | --- | --- | --- | --- |
|  |  | **Begin** | **End** | **Sequence** | **Begin** | **End** | **Sequence** |
| V$IKRS | Ikaros 1, potential regulator of lymphocyte differentiation | 39670 | 39682 | GGGGTTCCCATGC | 39790 | 39802 | GGGCTTCCCAGGC |
| V$AHRR | Aryl hydrocarbon receptor / Arnt heterodimers | 39762 | 39784 | AACAAGAGCACGCGCACCTGCCG | 39877 | 39899 | AACAAGAGCACGCGCACCTGTCG |
| V$CDEF | Cell cycle-dependent element, CDF-1 binding site (CDE/CHR tandem elements regulate cell cycle dependent repression) | 39767 | 39779 | GAGCACGCGCACC | 39882 | 39894 | GAGCACGCGCACC |
| V$HESF | Drosophila hairy and enhancer of split homologue 1 (HES-1) | 39771 | 39785 | ACGCGCACCTGCCGG | 39886 | 39900 | ACGCGCACCTGTCGG |
| V$MYOD | complex of Lmo2 bound to Tal-1, E2A proteins, and GATA-1, half-site 1 | 39771 | 39786 | ACGCGCACCTGCCGGC | 39886 | 39901 | ACGCGCACCTGTCGGC |
| V$ZBPF | Zinc finger transcription factor ZBP-89 | 39784 | 39806 | GGCCCGCCCGCCCCCTTGGTGCC | 39898 | 39920 | CGGCGCCCCGCCCCCTCCGCCCG |
| V$ECAT | nuclear factor Y (Y-box binding factor) | 39805 | 39819 | CCGGCCAATCGCGCG | 39919 | 39933 | CGGCCCAATCGTGGG |
| V$ZBPF | Core promoter-binding protein (CPBP) with 3 Krueppel-type zinc fingers | 39814 | 39836 | CGCGCGCTCGGGGCGGGGTCGGG | 39927 | 39949 | TCGTGGGCAGGGGCGGGGCCGGG |
| V$SP1F | stimulating protein 1 SP1, ubiquitous zinc finger transcription factor | 39820 | 39834 | CTCGGGGCGGGGTCG | 39933 | 39947 | GCAGGGGCGGGGCCG |
| V$MAZF | MYC-associated zinc finger protein related transcription factor | 39822 | 39834 | CGGGGCGGGGTCG | 39935 | 39947 | AGGGGCGGGGCCG |
| V$XBBF | X-box binding protein RFX1 | 39833 | 39851 | CGGGCGCGCTGGAACCAGA | 39947 | 39965 | GGGGCGCAGTCGGAACCCG |

**Human *SF1* Vs Mouse *Zfp162***

| **Family** | **Description** | **hgNM_004630 (Human SF1)** | | | **mgNM_011750 (Mouse Zfp162)** | | |
| --- | --- | --- | --- | --- | --- | --- | --- |
|  |  | **Begin** | **End** | **Sequence** | **Begin** | **End** | **Sequence** |
| V$PCAT | cellular and viral CCAAT box | 39907 | 39917 | GCAGCCAATGA | 39531 | 39541 | TCAGCCAATGA |
| V$ECAT | nuclear factor Y (Y-box binding factor) | 39907 | 39921 | GCAGCCAATGAGAGA | 39531 | 39545 | TCAGCCAATGAGCTA |
| V$RCAT | Mammalian C-type LTR CCAAT box | 39910 | 39934 | GCCAATGAGAGAGCTCGCCGTCGCT | 39534 | 39558 | GCCAATGAGCTAGCCGTTCTCTCAC |
| V$CREB | cAMP-response element-binding protein | 39926 | 39946 | GCCGTCGCTCCGTCATAGAGT | 39548 | 39569 | CGTTCTCTCACGTCACAGACT |
| V$MAZF | MYC-associated zinc finger protein related transcription factor | 39947 | 39959 | TCGCCCCACCCCA | 39652 | 39664 | CGGCCCCGCCGCC |
| V$SP1F | GC box elements | 39947 | 39961 | TCGCCCCACCCCATC | 39652 | 39666 | CGGCCCCGCCGCCAT |
| V$MAZF | Myc associated zinc finger protein (MAZ) | 39958 | 39970 | CATCCCCTCCTTT | 39652 | 39664 | CGGCCCCGCCGCC |
| V$XBBF | X-box binding protein RFX1 | 39986 | 40004 | AGTTCACGCAGTAACAAAT | 39642 | 39660 | CCAGTTCCCCCGGCCCCGC |
| V$CDEF | Cell cycle-dependent element, CDF-1 binding site (CDE/CHR tandem elements regulate cell cycle dependent repression) | 40006 | 40018 | AAGTGCGCGCTGC | 39602 | 39614 | CAGTCCGCGCAGT |
| V$CDEF | Cell cycle-dependent element, CDF-1 binding site (CDE/CHR tandem elements regulate cell cycle dependent repression) | 40006 | 40018 | AAGTGCGCGCTGC | 39621 | 39633 | GAGTGCGCGCTGC |
| V$ZF5F | Zinc finger / POZ domain transcription factor | 40008 | 40018 | GTGCGCGCTGC | 39621 | 39633 | GAGTGCGCGCT |
| V$GLIF | Zinc finger transcription factor GLI1 | 40019 | 40033 | GACACCTCCCAGCCC | 39634 | 39648 | GACACCTCCCAGTTC |
| V$VMYB | v-Myb | 40064 | 40074 | CCTAACGGTTC | 39544 | 39554 | TAGCCGTTCTC |
| V$VMYB | v-Myb | 40064 | 40074 | CCTAACGGTTC | 39679 | 39689 | CCTAACGGTTC |
| V$CMYB | c-Myb, important in hematopoesis, cellular equivalent to avian myoblastosis virus oncogene v-myb | 40064 | 40076 | CCTAACGGTTCGG | 39679 | 39691 | CCTAACGGTTCGG |
| V$DEAF | NUDR (nuclear DEAF-1 related transcriptional regulator protein) | 40070 | 40088 | GGTTCGGCCAATCCCAGCG | 39685 | 39704 | GGTTCGGCCAATCCCGACC |
| V$NFKB | NF-kappaB | 40072 | 40086 | TTCGGCCAATCCCAG | 39687 | 39701 | TTCGGCCAATCCCGA |
| V$PCAT | cellular and viral CCAAT box | 40073 | 40083 | TCGGCCAATCC | 39688 | 39698 | TCGGCCAATCC |
| V$ECAT | nuclear factor Y (Y-box binding factor) | 40073 | 40087 | TCGGCCAATCCCAGC | 39688 | 39702 | TCGGCCAATCCCGAC |
