# Supplementary material for: Microarray and comparative genomics-based identification of genes and gene regulatory regions of the mouse immune system
Source: BMC Genomics. 2004 Oct 25;5:82. doi: 10.1186/1471-2164-5-82 (PMC534115; doi:10.1186/1471-2164-5-82)
Supplement: Additional File 6 — CisMols display of location and composition of clusters of cis-elements that are putative regulatory modules for the genes in various groups (test and control). Each colored cube indicates a cluster of 3 or more cis-elements with at least one "lymphoid element". The region searched is upstream 3 kb and downstream 100 bp of transcription start site (as defined by the respective mRNAs from NCBI's RefSeq database). The legend in the lower left half of the figure indicates the composition of each of the modules and the genes that share them. [file 1471-2164-5-82-S6.pdf]

Hutton_HighThymus

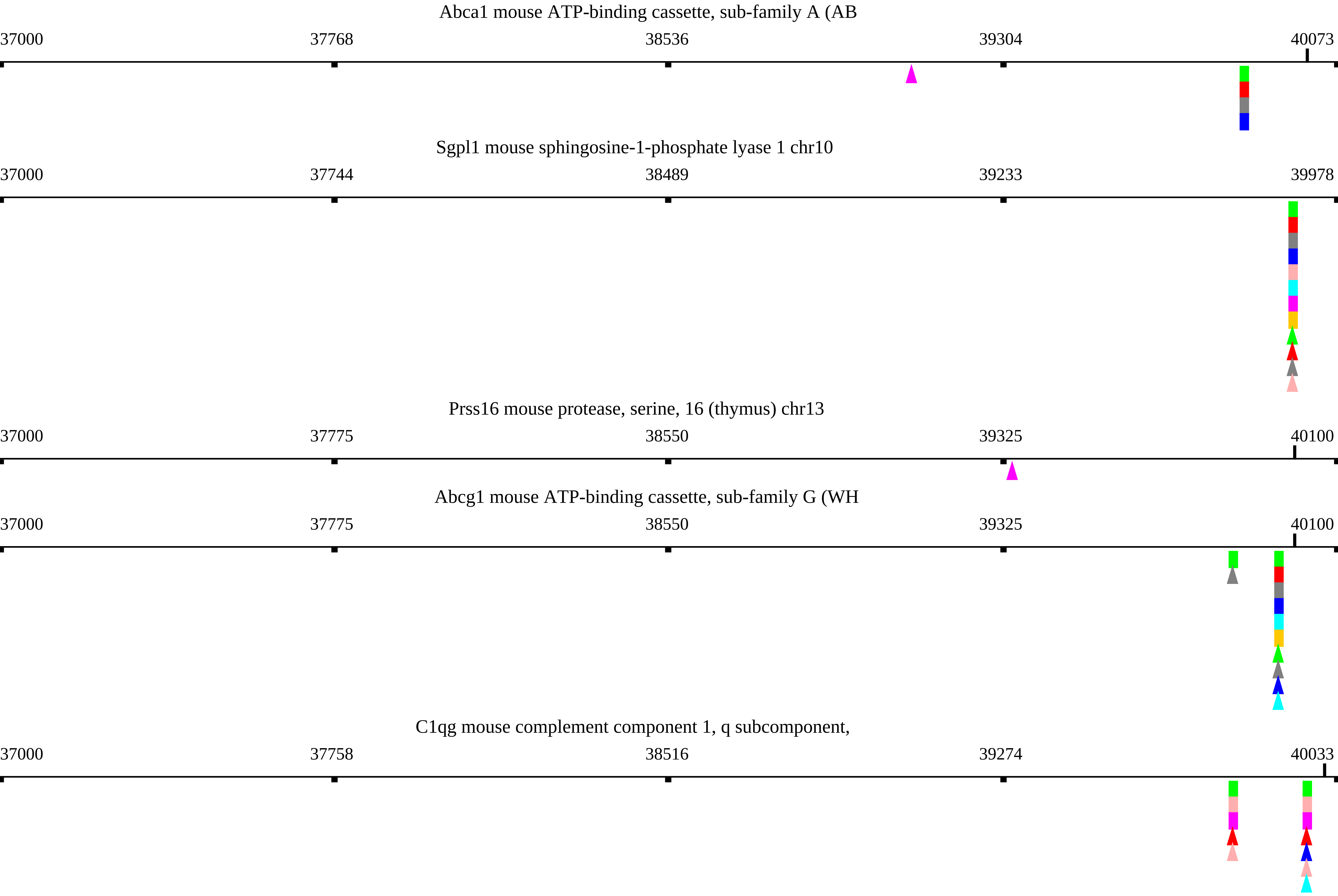

(c)2004 Cincinnati Children's Hospital Medical Center (http://cismols.cchmc.org)

		Genes with Cluster																
		4	3	3	3	3	2	2	2	2	2	2	2	2	2	2		
Genes	Sgpl1	X	X	X	X	X	X	X		X		X		X	X	X	12	Gene Frequency
	Abcg1	X	X	X	X	X	X	X	X			X	X			X	11	
	C1qg	X	X						X	X			X	X	X		7	
	Abca1	X		X	X	X					X						5	
	Prss16										X						1	
		Sites in Cluster																
Sites	V$SP1F	X	X	X	X	X	X	X	X	X		X	X	X	X	X	14	Site Frequency
	V$ZBPF	X	X		X	X	X	X	X			X	X	X	X	X	12	
	V$MAZF	X		X	X		X			X		X	X	X	X	X	10	
	V$EGRF			X	X	X		X				X				X	6	
	V$ETSF		X							X				X	X		4	
	V$AP2F							X				X				X	3	
	V$HESF						X					X					2	
	V$MZF1								X				X				2	
	V$STAT													X			1	
	V$HAML										X						1	
	V$MYT1										X						1	
	V$GATA										X						1	
		3	3	3	4	3	4	4	3	3	3	6	4	5	4	5		
		Sites in Cluster																
